# Mutation Bias Favors Protein Folding Stability in the Evolution of Small Populations

**DOI:** 10.1371/journal.pcbi.1000767

**Published:** 2010-05-06

**Authors:** Raul Mendez, Miriam Fritsche, Markus Porto, Ugo Bastolla

**Affiliations:** 1 Centro de Biología Molecular “Severo Ochoa”, Consejo Superior de Investigaciones Científicas and Universidad Autónoma de Madrid, Madrid, Spain; 2 Institut für Festkörperphysik, Technische Universität Darmstadt, Darmstadt, Germany; Harvard University, United States of America

## Abstract

Mutation bias in prokaryotes varies from extreme adenine and thymine (AT) in obligatory endosymbiotic or parasitic bacteria to extreme guanine and cytosine (GC), for instance in actinobacteria. GC mutation bias deeply influences the folding stability of proteins, making proteins on the average less hydrophobic and therefore less stable with respect to unfolding but also less susceptible to misfolding and aggregation. We study a model where proteins evolve subject to selection for folding stability under given mutation bias, population size, and neutrality. We find a non-neutral regime where, for any given population size, there is an optimal mutation bias that maximizes fitness. Interestingly, this optimal GC usage is small for small populations, large for intermediate populations and around 50% for large populations. This result is robust with respect to the definition of the fitness function and to the protein structures studied. Our model suggests that small populations evolving with small GC usage eventually accumulate a significant selective advantage over populations evolving without this bias. This provides a possible explanation to the observation that most species adopting obligatory intracellular lifestyles with a consequent reduction of effective population size shifted their mutation spectrum towards AT. The model also predicts that large GC usage is optimal for intermediate population size. To test these predictions we estimated the effective population sizes of bacterial species using the optimal codon usage coefficients computed by dos Reis *et al.* and the synonymous to non-synonymous substitution ratio computed by Daubin and Moran. We found that the population sizes estimated in these ways are significantly smaller for species with small and large GC usage compared to species with no bias, which supports our prediction.

## Author Summary

The Guanine plus Cytosine (GC) content of bacterial genomes varies from 20% to 80%. This variation is attributed to the mutation bias produced by replication and repair machinaries. However, the evolutionary forces that act on these very different machinaries have remained elusive. It is known that the GC content of genes strongly influences the resulting proteins' hydrophobicity, which is the main determinant of folding stability. This may lead to expectation that the GC content is strongly selected at its optimal value, since proteins that are too hydrophylic face unfolding problems and proteins that are too hydrophobic face misfolding and aggregation problems. In this work, using a realistic model of genotype (DNA sequence) to phenotype (protein folding stability) to fitness mapping and a standard population genetics model, we find that the optimal GC usage depends on population size. In particular, very small populations prefer small GC usage, intermediate populations prefer large GC usage, and large populations prefer no bias. Our results may explain why most intracellular bacteria, evolving with small effective populations, tend to adopt small GC usage. To test this hypothesis, we estimated the effective population size of several bacterial species, finding that those that evolve with 50% GC usage are characterized by significantly larger populations, although several exceptions exist.

## Introduction

The quantitative modeling of molecular evolution is of key importance for reconstructing evolutionary histories, as well as for understanding how the properties of natural macromolecules are influenced by their evolution. Already for a long time population size has been recognized as a crucial factor that influences both the evolutionary process and the stability that macromolecules can attain. On the other hand, even if mutation bias in prokaryotes varies from extreme GC rich to extreme AT rich, its influence on the evolutionary process, the stability of evolving macromolecule, and on the fitness of the population has received much less attention. Here, we simulate an evolutionary model that combines population size, GC mutation bias, and protein folding stability, and we show the deep interplay between these variables.

Kimura's neutral model [1], [2] is still one of the most influential models of molecular evolution. This model considers all viable macromolecules as equally fit and all the others as nonviable. Within this neutral model, the functional properties of the evolving macromolecules, in particular their folding stability, are independent of population size and, by entropy arguments, they are expected to coincide with the minimal properties compatible with viable molecules [3]. If mutations with small fitness effects are included in the model, population size 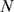 becomes a key variable of the evolutionary process, since slightly deleterious mutations are more likely to be fixed in small populations [4]–[6]. This study has been pioneered by Ohta, who showed that population size can provide a possible explanation for empirical observations such as the generation time effect [7], [8]. Obligate intracellular lifestyle, such as that of endosymbiotic or parasitic bacteria, implies a strong reduction in effective population size due to bottlenecks upon transmission from one host to another. Inspired by Ohta's theory, computational studies have compared bacterial species displaying an obligate intracellular lifestyle with their free living relatives, suggesting that the genes of intracellular bacteria evolve faster as a result of relaxed selection [9] (but Itoh *et al.* [10] give a different interpretation) and that their structural RNAs [11] and their proteins [12] are less stable than the orthologous macromolecules of free living bacteria. Evolution experiments with virus and bacteria confirm the influence of small population size, demonstrating fitness loss in populations evolving under repeated bottlenecks [13], [14], and show that such a loss can be partly compensated by over-expressing chaperones that assist protein folding [15]. These findings support the idea that fitness is reduced in small populations as a consequence of the reduction of protein folding stability. Recent theoretical work has shown that, in the appropriate limits, the statistical properties of population genetics are formally equivalent to a statistical mechanical system, so that there is an exact analogy between the reduction of fitness for small populations and the increase of entropy for large temperature [16], [17]. In the present study, we will exploit this correspondence to get analytic insight into non-neutral evolution.

Another key evolutionary variable, which however has received little attention, is the nucleotide spectrum. In prokaryotic genomes, it varies from extreme adenine plus thymine (AT) content in obligatory intracellular bacteria to extreme guanine plus cytosine (GC) content, for instance in actinobacteria. These differences in GC content are prevalently thought to be due to mutation bias [18], [19]. They are strongest at the third codon position, where GC content barely affects the amino acid composition of the protein, but also influence the coding positions [20], [21]. Due to the structure of the genetic code, a mutation bias favoring thymine at the nucleotide level favors the incorporation of hydrophobic amino acids in the translated protein [12], [22]. Hydrophobicity is a key property for protein folding [23]. Proteins that are too hydrophylic tend to be naturally unfolded, whereas proteins that are too hydrophobic tend to misfold and aggregate [24]. This qualitative trade-off between unfolding and misfolding was confirmed by a computational study of the properties of homologous proteins in the proteomes of several bacterial species, using a model of protein folding stability that correlates well with experimentally measured unfolding stabilities [12]. In previous work, two of us and colleagues investigated the relationship between unfolding stability, misfolding stability and mutation bias using a protein evolution model with a realistic genotype (DNA sequence) to phenotype (folding stability) mapping in a neutral fitness landscape in which all proteins with stabilities above thresholds have the same fitness. We found that the mutation bias modulates the trade-off between the two kinds of stability, making proteins evolving under AT mutation bias more stable against unfolding but less stable against misfolding [25].

Interestingly, the two aspects discussed above, small population size and mutation bias towards AT, are strongly correlated in nature. In fact, most bacterial and eukaryotic lineages that adopted an intracellular lifestyle, with consequent reduction of their effective population size, also shifted their mutation spectrum towards AT [26], as indicated by the strong correlation between reduced genome size, which is a signature of intracellularity, and the AT bias [9], [12]. In this work, we investigate the association between population size and mutation bias, studying its consequences through a model that takes into account all of the relevant features of protein evolution discussed above: folding stability with respect to both unfolding and misfolding, population size, mutation bias, and neutrality, i.e. the relationship between folding stability and fitness.

## Results

### Model

We adopt the Moran model [27], which describes an evolving haploid population with 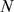 individuals that reproduce asexually and stochastically under mutation and selection. The model can be easily extended to diploid populations. We assume here that the product of population size times mutation rate is small, 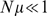, so that the population is monomorphic, i.e. the time scale for appearance of a new mutant in the population is large and at most one single mutant genotype is competing with the wild-type for fixation each time. This assumption is justified for small and intermediate populations when considering an individual protein coding gene, but not an entire genome (see Discussion). However, for large populations the assumption 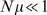 is violated even for an individual gene, and we can not apply the model to this case. In this monomorphic limit, the probability that a mutation arising as a single individual is fixed in the whole population can be exactly computed as [27]

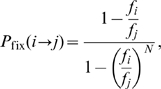
(1)where 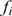 is the exponential growth rate of the phenotype associated to sequence 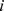, which will be called fitness in the following. This analytic result enormously simplifies the numeric study of the system allowing the systematic exploration of its parameter space. In our simulations, we randomly generate a mutated sequence, evaluate its fitness with respect to the wild type, and accept the new mutation according to the above probability.

We model mutations at the DNA level through the HKY process [28], whose only parameters are the equilibrium frequencies of the four bases 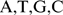 in the absence of selection, and the transition/transversion ratio 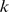, whose influence is very weak and which we set to 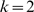 [8]. In order to reduce the number of parameters, we assume that Chargaff's second parity rule holds, so that 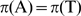 and 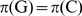. Thus, the mutation model only depends on the GC usage, 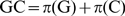. GC usage different from 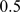 determines a mutation bias towards AT or towards GC, therefore we sometimes refer to the GC usage variable as the mutation bias. In our model, the GC usage variable very strongly correlates with the GC content of the evolving gene in the stationary state of the evolutionary dynamics. The same correlation is thought to exist between the GC content of bacterial genomes, in particular at third codon position, and the GC usage of the mutations arising in bacterial replication. Therefore, we will compare the variable GC usage in our model with the variable GC content at third codon position in bacterial genomes.

#### Folding stability

In our model the fitness of an individual carrying a particular gene depends on the folding properties of the translated protein, which are estimated through a simple protein folding model. This model was used in our previous works [25], [29], [30] and it is similar to those used by others [31]–[39]. A characteristic of our model that distinguishes it from similar ones is that we consider two types of stability, with respect to misfolding and with respect to unfolding. Stability with respect to unfolding is estimated through the folding free energy 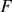 of a protein sequence 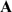, calculated with a simple contact interaction model (see Methods). Free energies estimated in this way correlate well with experimental measures (correlation coefficient 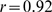 over a test set of 20 proteins, UB, unpublished result). Stability with respect to misfolding is estimated through the normalized energy gap 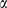 (see Methods), which is the normalized difference between the effective energy of the native state and the minimum effective energy predicted through a Random Energy Model, representing the energy of compact intermediate structures very different from the native one. These misfolded structures can trap the folding process, and they can expose hydrophobic patches and promote aggregation.

Interestingly, these two kinds of stability respond in an opposite way to an increased mutation pressure towards hydrophobicity: while 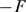 increases for increasing mean hydrophobicity, meaning that proteins become more stable with respect to unfolding, the normalized energy gap decreases. This is due to the fact that the maximum stability of all potential misfolded structures increases more than the stability of the native structure, thus making misfolding and aggregation problems potentially more serious [12]. This trade-off between the two stabilities has a deep influence on the evolutionary dynamics.

#### Fitness

**Figure 1:**
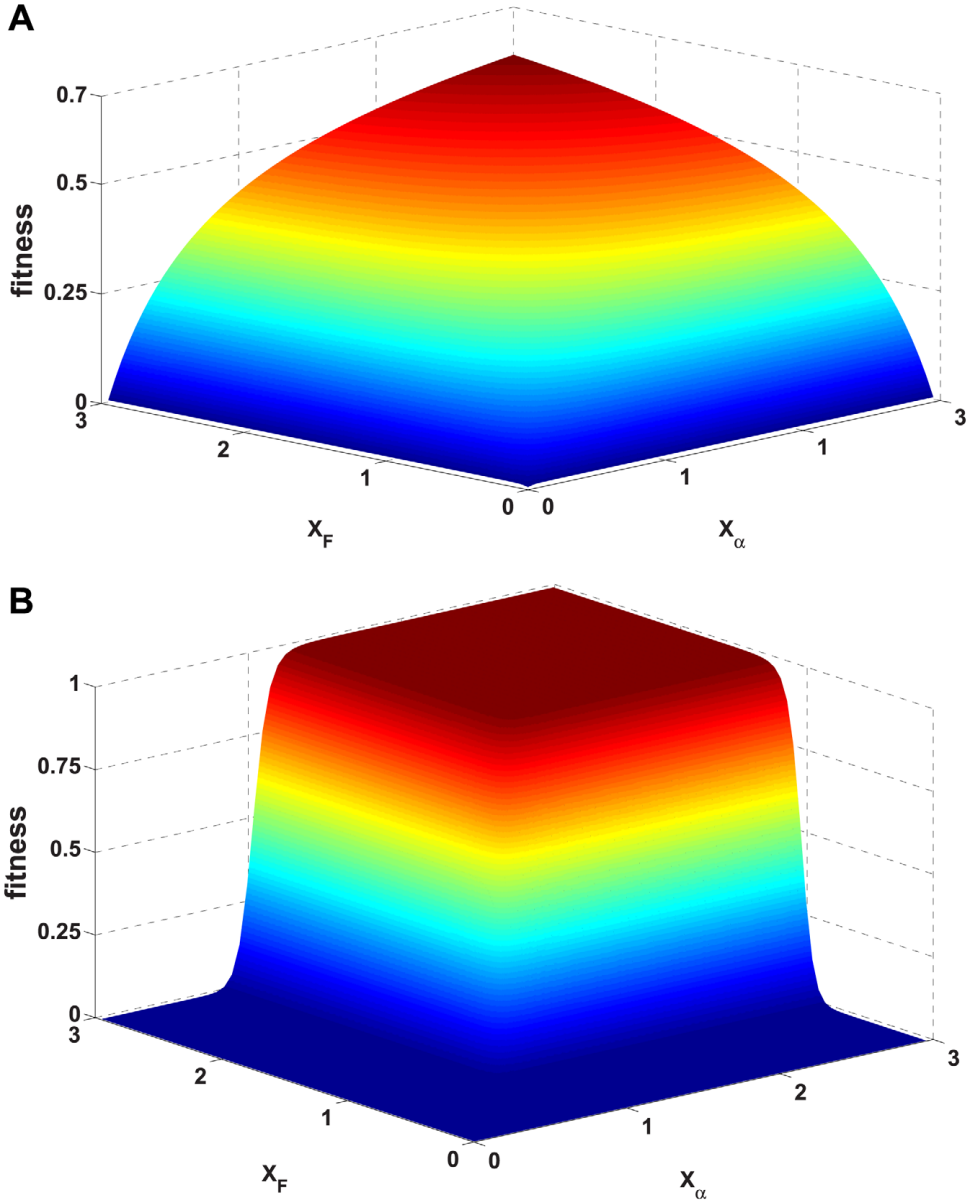
Fitness versus stabilities for 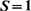 (top) and 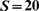 (bottom).

We adopt a fitness function that depends on the normalized stabilities 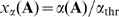 and 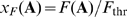 and on the neutrality exponent 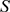,
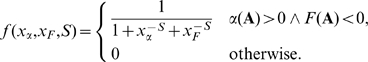
(2)The neutral thresholds 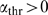 and 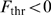 define the scale of acceptable stabilities and they are kept fixed throughout the simulation. With this definition the fitness takes values between 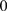 and 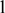, vanishing if the protein does not fold correctly, which means that it is considered essential. Two plots of fitness versus stability for 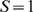 and 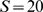 are represented in Fig. 1 for illustration purposes. The fitness becomes a binary variable, either 0 or 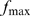, if the neutrality exponent 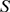 is either zero (in this case all sequences satisfying 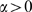 and 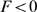 are equally fit) or infinite (in this case all sequences overcoming the neutral thresholds 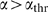 and 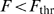 have fitness 1 and all other sequences are not viable). These limits are equivalent to Kimura's neutral model [2], which we studied previously [25], [29], [30], in which it is assumed that mutations that maintain stabilities above the neutral thresholds have no fitness effect, while all the others are lethal. This motivated us to name the parameter 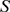 the neutrality exponent. Notice that the term neutrality is sometimes defined as the fraction of proteins that retain wild-type structure under mutations [40]. This definition assumes a neutral model where the wild-type structure is either stable (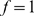) or unstable (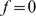). We prefer to call this quantity the fraction of neutral neighbors [29], and to call neutrality exponent the exponent 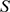 that determines the smoothness of the relationship between stability and fitness.

We choose the two neutral thresholds proportional to the values of 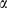 and 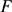 for the reference protein in the Protein Data Bank (PDB), multiplied with coefficients 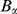 and 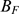. In simulations of neutral evolution, 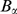 and 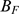 have to be smaller than one so that the reference protein is viable. We present results with 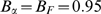. We tested the robustness of our results with respect to both changes in the analytical form of the fitness function and the values of parameters, as discussed in the following.

### Analytic results

We can analytically predict how the population size 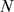 and the neutrality exponent 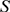 influence stability and fitness by exploiting the formal analogy between population genetics and statistical mechanics demonstrated by Berg and coworkers [16] and by Sella and Hirsh [17]. These authors noticed that, in the monomorphic limit 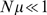 mentioned above and that we assume throughout this work, the Moran process, as well as other evolutionary processes studied in population genetics, tends to a stationary distribution of the form 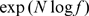. This distribution is equivalent to a Boltzmann distribution where population size 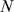 plays the role of inverse temperature and the logarithm of fitness, 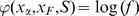 plays the role of minus energy. This result implies that the probability to find a protein with stability values 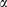 and 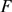 in the stationary state of an evolving population is proportional to 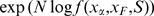 multiplied by a factor that depends on the mutation process. The bias arising in the mutation process was treated as a “chemical potentia” by Sella and Hirsh [17] or as a mutational entropy by Berg et al. [16]. These two formalisms are qualitatively equivalent. We find the name mutational entropy more intuitive, and we will use it in the following. We define 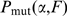 the probability to find stability parameters 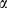 and 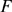 under mutation alone, and we introduce the quantity 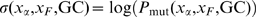, which we call the mutational entropy compatible with stabilities 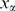 and 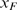 under the given mutation process (notice that strictly speaking 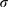 is not an entropy, however we find this name intuitive for indicating the mutational force that opposes protein stability). As discussed above, the mutational entropy depends on the GC usage, which can favor one kind of stability with respect to the other. Taking all this into account, the stationary distribution of stability that results from mutation and selection is
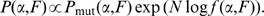
(3)The logarithm of the above probability can be interpreted as minus an evolutionary free energy divided by temperature 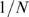, and it is given by
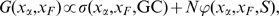
(4)where 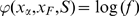 is called the additive fitness [17]. The distribution Eq. (3) is peaked around the values 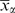 and 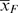 that maximize the exponent 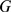, i.e. minimize the evolutionary free energy. The equations that define these most likely values read
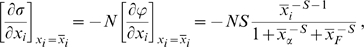
(5)where 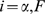. We call the above the maximum-likelihood (ML) equations. Notice that the maximum likelihood values 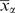 and 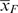 depend on the parameters 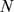, 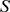 and 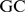. We can study this dependence analytically, assuming that Eq. (3) is narrowly peaked around these values, so that averages can be calculated as 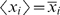 and 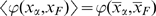. This approximation is justified by the fact that the mutational entropy 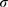 is expected to be proportional to protein length 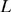, which is of the order of 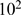, and the selective term is proportional to population size, which is also large, so that the exponent 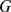 is large and the distribution very narrow. The condition that 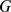 has a maximum at 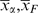 requires that its Hessian matrix 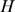, consisting of its second derivatives, is negative definite,
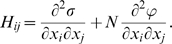
(6)This Hessian is the sum of the Hessian of 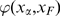, which is negative by construction, as it is easy to verify, and the Hessian of 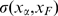, which is the logarithm of a probability. We assume that the mutational entropy 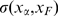 has a single maximum at stabilities 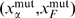, so that its Hessian is negative. The values 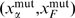 that represent the most likely values of 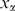 and 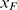 in the absence of selection depend on 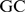. By definition of 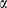, 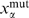 is always negative, which is not a viable stability (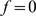). However, our numerical results show that 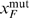 is positive for small GC usage, corresponding to hydrophobic sequences. The mutational entropy 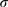 decreases for 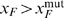 and for 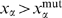, which implies that the corresponding derivatives are negative, as required for the existence of the solution of the ML equations.

We can go beyond the maximum-likelihood approximation writing the exponent 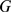 at second order as 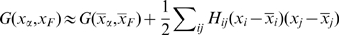, which is equivalent to approximating the distribution Eq. (3) as a Gaussian with covariance matrix 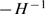. Therefore, negativity of the Hessian matrix is equivalent to requiring the covariance matrix to be positive.

#### Influence of population size

We can calculate how 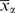 and 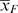 depend on population size by taking the derivatives of the ML equations with respect to 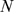 (see Text S1). In this way, we find that both stabilities must increase with population size, as expected. The mean fitness 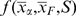 is therefore an increasing function of 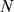, whereas the mutational entropy 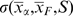 is a decreasing function of 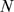.

#### Influence of the neutrality exponent

Stabilities are not monotonic functions of the neutrality exponent 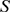. At 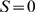 all stabilities above the lethal threshold 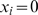 at which fitness drops to zero are selectively equivalent, and the ML equations imply that the stabilities with the largest mutational entropy fulfilling these conditions will prevail. As mentioned above, the most likely value of 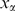 in the absence of selection is negative for all 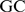 usages, so that 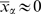 for 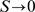. On the other hand, the most likely value of 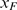 in the absence of selection 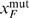 is positive for hydrophobic sequences, corresponding to small GC usage. The ML equations thus predict that 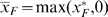, where 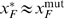 satisfies the equation 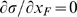 at 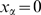. Similarly, in the neutral limit 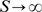, the smaller between 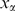 and 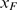 tends to the value 1, i.e.the corresponding stability tends to the neutral threshold, and the larger stability satisfies the equation 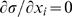 at 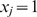. For finite 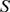, it can be shown that both stabilities increase with 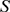 when 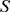 is small, they reach a maximum and then decrease towards the neutral values (see Text S1). This behavior of stability arises from the fact that, under neutral or almost neutral evolution, the advantage in fitness provided by a more stable protein is too small to be fixed in the population against the entropic effect of mutations. This mechanism has been proposed as an explanation of the empirical observation that natural proteins are only marginally stable [3].

Similarly, we can show that the fitness has a minimum as a function of 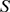: It starts from the value 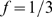 at 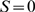, then at small 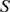 the fitness is reduced because low stability values are penalized, at larger 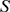 more stable sequences are attained, and finally in the neutral limit the fitness tends to the maximum possible value 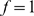 while stability decreases (see Text S1). We can therefore distinguish three qualitative behaviors, described in Table 1. We are mainly interested in the parameter range that is far both from the region 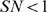 at which the minimum stability is close to the lethal threshold 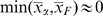, and from the region of large 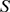 at which stabilities are close to the neutral thresholds.

**Table 1:** Qualitative behavior of fitness and stability versus neutrality exponent 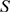 at fixed GC and population size.

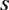 range	Stability	Fitness
Small	Increasing	Decreasing
Intermediate	Increasing	Increasing
Large	Decreasing	Increasing

At 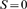 stability is close to the lethal threshold 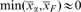 without any penalization for the fitness. In the small 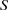 regime stability increases with 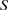, but the penalization for low stability decreases even more, with the net effect of a decrease in fitness. At intermediate 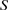 both stability and fitness increase with 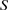 and stability reaches a maximum that depends on 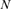. Finally, at large 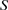 stability decreases with 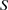, since the differences in fitness produced by a given difference in stability become smaller and cannot be fixed against the entropic effect of mutations, while fitness tends to the maximum possible value 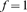.

#### Influence of the mutation bias

The most interesting feature of the evolutionary model presented here is the dependence of stability and fitness on the mutation bias. Unfortunately, this dependence cannot be predicted analytically, since we do not have a detailed model of how the mutation entropy 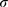 depends on GC usage. Numerical results show that, for the folding free energy function that we adopt here, the two stabilities respond differently to the GC usage. This is expected, since small GC usage favors hydrophobic proteins, enhancing unfolding stability (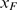) at the expenses of misfolding stability (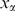). Since fitness depends on both 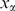 and 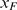, it has to trade-off between the two stabilities, and we expect that there is an optimal GC usage at which the fitness is maximal for given 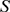 and 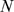, which satisfies the equation 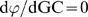


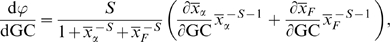
(7)where 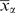 and 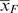 are determined by the ML equations (5). The maximum fitness is achieved when the quantity
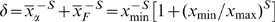
(8)is minimal. Here 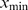 is the smaller value and 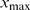 the larger value of 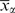 and 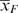. We first discuss the small 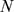 regime at which stabilities are small and they are strongly influenced by the GC usage. In this regime, we expect that there is a value of 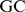 at which 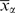 and 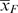 are equal. Therefore, at small 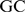 usage it holds 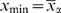, which increases with 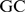, whereas at large 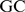 usage it holds 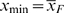, which decreases with 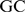. Consequently, the factor 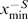 has a minimum where 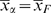. Conversely, the second factor that appears in 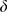, 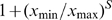, has a maximum where 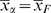. We expect that the factor 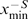 depends more strongly on 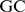 than the factor 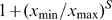, in particular if 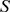 is large. Therefore, we expect that the minimum 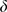 (i.e. the optimal 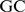) is reached near the 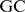 usage at which 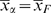, and that it approaches this value as 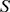 grows. The 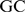 usage at which 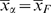 has an interesting interpretation. We can define the selective pressure on the variable 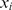 as the derivative of 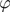 with respect to 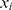, which expresses how fitness responds to a change in stability. If this derivative is large, a large number of attempted mutations will be discarded because of their negative influence on fitness. The ML equations show that the selective pressure is proportional to 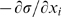, and it is stronger on the smaller variable 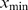. Therefore, when the 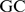 usage increases, the selective pressure on unfolding increases, and the selective pressure on misfolding decreases, and they balance when 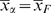.

Theoretical considerations and numerical results indicate that there is a second regime at large 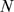. In this limit, the fitness tends to the maximum possible value. Due to the trade-off between unfolding and misfolding stability, it is not possible to maximize 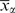 and 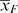 simultaneously, since they are inversely related. As 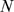 increases, 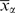 and 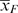 are expected to converge to the optimal fitness point 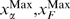 and their dependence on 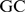 is expected to become weaker and weaker. We find numerically that 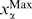 is smaller than 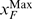, so that for large 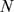, 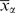 is smaller than 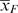 for all 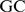, and the selective pressure is always stronger on 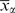. In this regime, 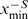 always decreases with 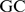 and its dependence on 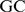 gets weaker. Conversely, the term 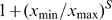 always increases with 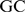, and the optimal 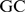 is determined by a balance between these two terms. We now discuss two interesting limiting behaviors of the optimal 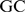.

1. In the small 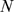 regime and for finite 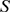, so that 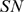 is small, 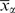 tends to zero and 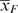 tends to 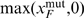 independent of 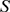. For small GC usage, 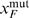 is positive and 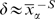 is a decreasing function of GC, since 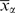 increases with GC. For large GC usage, 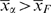 and 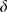 increases with GC. Therefore, we expect that the minimum of 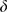, i.e. the optimal GC, is attained near the GC usage at which 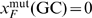, which is independent of 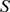 and of the neutral thresholds 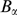 and 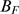.
2. In the neutral limit 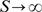, the selective pressure only affects the smallest stability variable, since 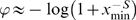. This tends to 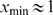 independent of 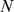 and 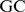. Therefore, as discussed above, for large 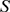, the optimal 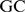 is reached when 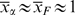, i.e. when the two selective pressures balance. The ML equations imply that at this point 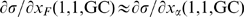, so that the optimal 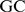 does not depend on 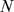. The ML equations also imply that, in the large 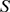 limit, 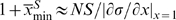 (see Text S1), which means that the maximum stability and maximum fitness is attained at the 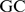 value at which 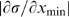 is minimum. This prediction is confirmed in Fig. 6 in the Text S1).

### Simulations

**Figure 2:**
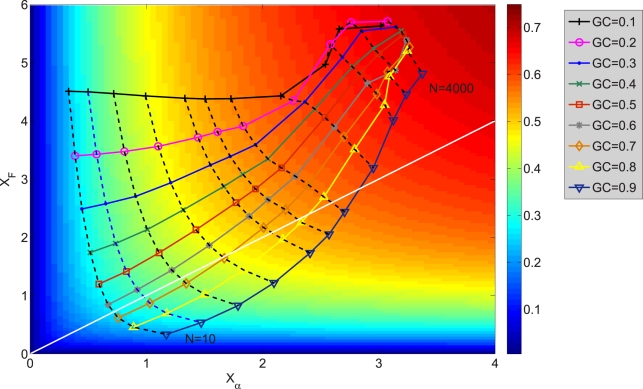
Mean unfolding stability 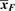 versus misfolding stability 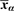 for neutrality exponent 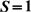 (non-neutral regime). The sets of points joined with solid lines correspond to constant GC usage, between 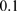 (largest 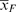) and 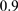 (largest 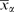). 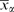 grows and 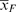 decreases with 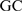. The sets of points joined with dashed lines correspond to constant population size 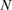, from 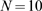 (smallest stability) to 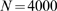 (largest stability). Both stability variables 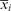 increase with 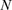. Data points are superimposed to a heat map of the fitness function, showing that fitness increases with 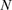. However, constant 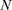 lines do not correspond to constant fitness, but there are small variations, from which the optimal GC usage is derived. The solid white line shows 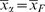 at which the selective pressures on 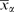 and 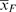 balance. One can see that, at large 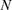, 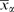 is smaller than 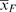 for all 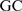, so that the selective pressure is stronger on the former.

All simulations presented here are based on the native structure of some natural protein. When not otherwise stated, we exemplify our numerical results using the protein lysozyme, PDB id. 31zt. In all cases, the starting sequence is the sequence in the PDB. Results are collected after fitness has converged to its stationary value, discarding the first 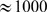 accepted substitutions, which are enough for equilibration, as it can be seen in Fig. 2 in the Text S1.

As an illustration of the stationary states of the evolutionary dynamics, we represent in Fig. 2 the mean stability values 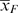 and 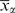 obtained using the fitness function with 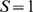 for different population sizes from 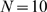 to 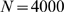 and GC usage from 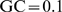 to 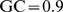. The distributions 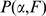, Eq. (3), are narrowly peaked around the plotted points 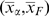. Sets of points with the same GC usage are joined with solid lines, and sets of points with the same 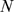 are joined with dashed line. The data are superimposed to a heat map that shows the value of fitness in colour code. We can see from the figure that both stabilities grow with 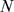. On the other hand, 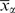 grows and 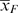 decreases with 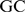, so that 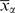 and 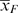 are negatively correlated for fixed population size. For 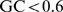, 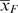 tends to a finite value when 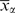 tends to zero (corresponding to very small 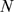), i.e. the most likely value of 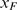 in the absence of selection is 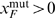 and, for such small GC usage, there is very weak selective pressure on unfolding. One can see from the plot that the GC usage at which 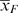 and 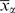 are equal increases with population size, which implies that the selective pressure on 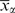 increases more than the selective pressure on 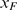 for increasing population size. In the large population limit both 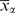 and 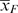 tend to finite values independent of GC. We estimated from our numerical results that 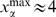 and 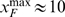, so that for large populations it is always 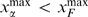.

**Figure 3:**
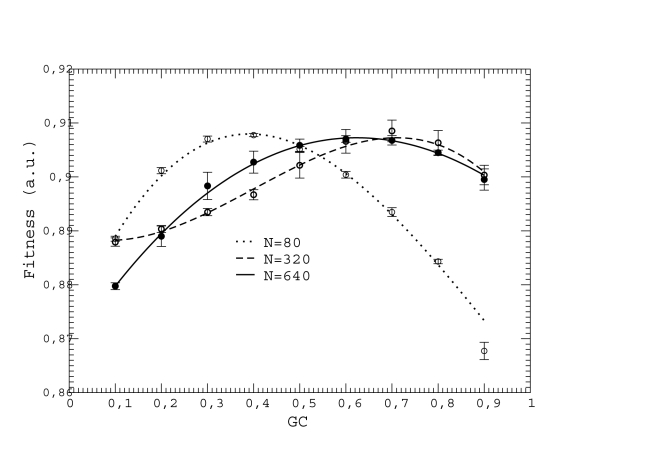
Fitness (in different units for each curve) versus GC usage for neutrality exponent 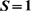 and three different population sizes. The curves have been shifted in the vertical direction so that their maxima coincide. We obtain 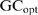 by cubic fits, which are plotted as dotted, dashed, and solid lines.

**Figure 4:**
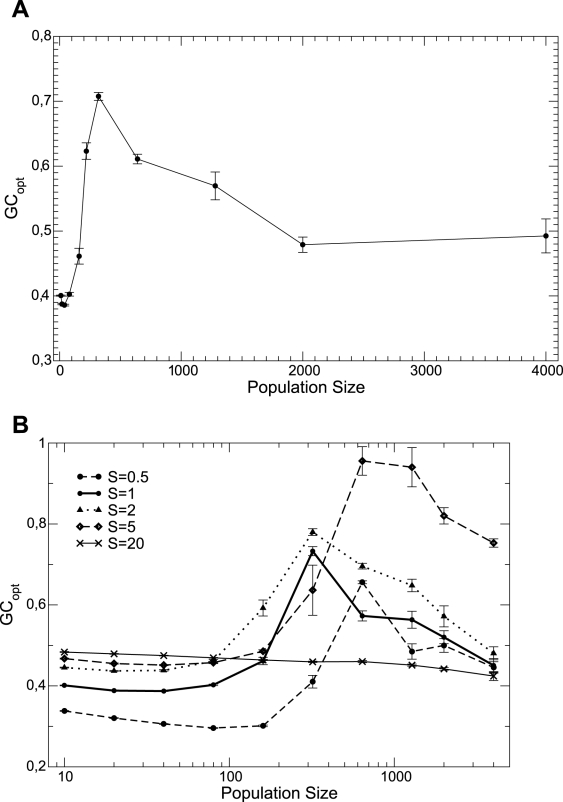
Optimal GC usage 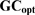 at which the fitness is maximum versus population size 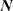. The upper plot shows data with neutrality exponent 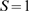 and the bottom plot shows 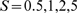 and 20. Interpolating lines are drawn as a guide to the eye.

Fitness clearly increases with 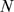. The variation of fitness with 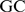 is weaker, but one can nevertheless notice it from the plot. This variation translates into the fact that, for fixed fitness function and population size 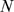, there is an optimal 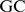 usage such that fitness is maximal, as predicted in Eq. (7). The existence of this optimal mutation bias is demonstrated in Fig. 3, where we plot the fitness of populations with constant 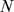 and 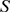 as a function of their 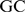 usage. For each set of parameters, we obtained the optimal GC usage 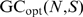 by cubic interpolation, as exemplified in Fig. 3, and plotted it versus 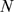. We found that 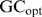 is small for very small populations, large for intermediate populations, and the bias is almost absent (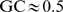) for very large populations (see Fig. 4). We obtained qualitatively similar results as long as the neutrality exponent 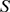 is not too large or too small (in that case, the fitness landscape becomes almost neutral). The population size at which the optimal GC usage is highest increases with decreasing 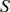 for small 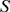, while the opposite holds for large 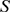. Our numerical results are consistent with the optimal GC usage becoming less dependent on 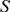 in the infinite population limit, see Fig. 3 in the Text S1.

Eq. (4) implies that a trait that confers a selective advantage can only be fixed against the entropic effect of random mutations when the difference in the selection coefficients 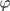 is larger than 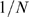. We therefore verified whether the difference of selective coefficients 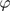 between populations adopting different GC usages is large enough so that the optimal one would be eventually selected. We found that 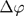 decreases with population size, but more slowly than 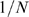, so that 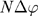 increases with 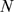, see Fig. 4 in the Text S1. This implies that two populations evolving with different mutation bias (the optimal one and another one) attain a fitness difference large enough so that the optimal GC usage can be selected.

**Figure 5:**
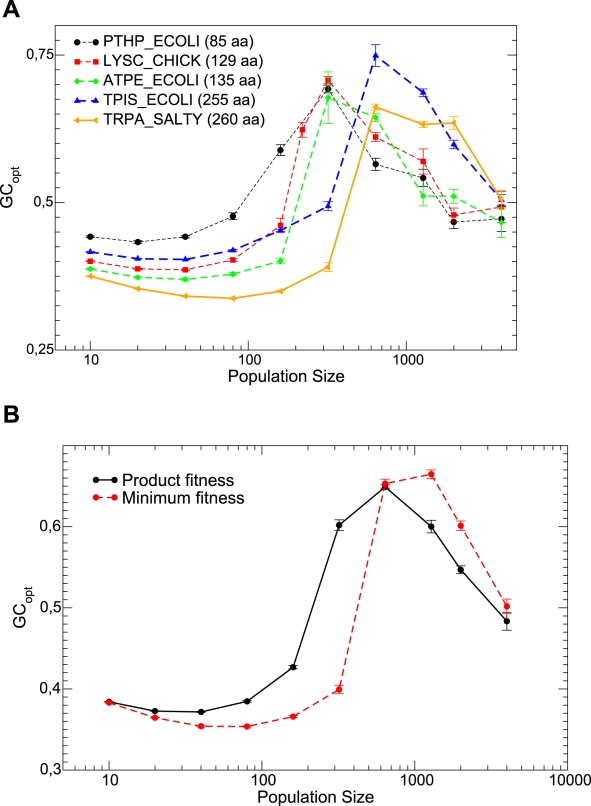
Optimal mutation bias 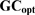 at which the fitness is maximum versus population size 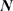 for different proteins and neutrality exponent 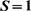. Upper plot: Results for individual proteins. Bottom plot: Fitness is obtained for the combination of 5 proteins either as the minimum or as the product over all proteins. Interpolating lines are drawn as a guide to the eye.

We tested that our results do not change qualitatively when different protein structures are used in the simulation. To this end, we computed the relationship between the optimal GC usage and population size at neutrality exponent 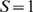 for five proteins of different length and secondary structure (see Methods). All curves, plotted in Fig. 5, have the same shape, although they are shifted in the vertical direction in a way that suggests that shorter proteins are characterized by larger optimal GC usage (but more proteins are needed to confirm this trend). We then combined the five curves. We assumed that a genome composed of these five proteins is evolving with very low mutation rate, so that at most one protein is mutated at each step, consistent with the assumption 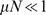. The global fitness of the organism was obtained through two different ansatz that yielded qualitatively similar results, either as the minimum of the fitness of all proteins 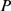, 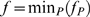 or as the product of the fitnesses, 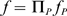, assuming absence of epistatic interactions. From these 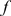 we then obtained the optimal GC by cubic interpolation. This is represented in Fig. 5, bottom plot for 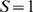. One can see that the qualitative behavior of the individual curves is preserved. We expect therefore that this qualitative behavior would be maintained for a large number of proteins as well.

**Figure 6:**
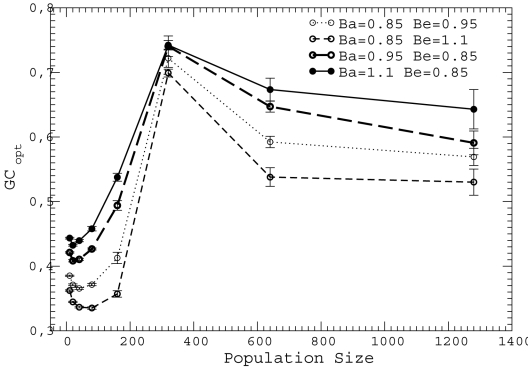
Optimal GC usage 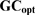 versus population size 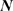 for neutrality exponent 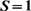 and different values of the neutral thresholds 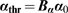 and 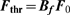, where the reference energy gap 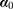 and unfolding free energy 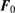 are those measured for the protein in the PDB. We simulated all nine combinations of the values 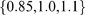 for either 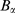 of 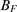. We only show four combinations since all other curves are contained between them.

To further test the robustness of our results we changed the neutral thresholds 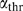 and 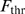 up to 20%, examining nine combinations of thresholds for neutrality exponent 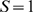. The results are shown in Fig 6. One can see that the qualitative behavior is unchanged. As expected, when 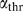 becomes more tolerant the optimal GC usage decreases, and the contrary happens when 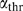 becomes more strict.

**Figure 7:**
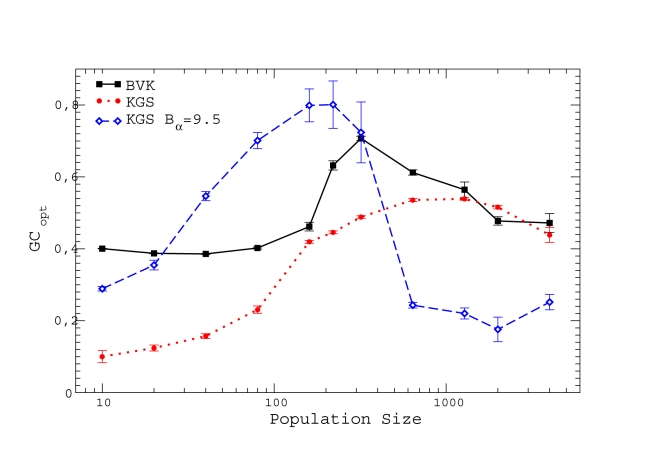
Comparison between the optimal GC usages computed with GKS energy parameters (dotted line and dashed line) and the BVK parameters adopted in the present study (solid line). The conformation entropy is 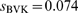 for BVK parameters and 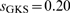 for GKS. The coefficient of the neutral threshold is 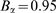 for the dotted curve and 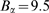 for the dashed curve. Other parameters are fixed at 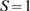, 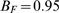.

Finally, we verified that the results are robust with respect to the energy parameters used. For such a test, we adopted the contact interaction energies determined by Godzik, Kolinsky and Skolnick (GKS) [41]. These parameters have correlation 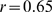 with the BVK parameters adopted in the present study, so that their differences are not small. We determined a new parameter for conformation entropy 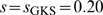 by demanding the folding free energies computed with the two sets of energy parameters to coincide on the average. As one can see from the dotted curve in Fig. 7, the qualitative behavior is the same for the two parameter-sets, but the optimal GC usage for GKS parameters is lower than for BVK parameters. This is due to the fact that, for our test protein lysozyme, GKS energy parameters produce a very low normalized energy gap 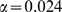 instead of 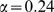 with BVK parameters, which means that the native conformation is closer in energy to random conformations when GKS parameters are used. Consequently, 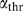 is very small (we recall that 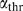 is proportional to the value of 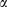 for the native sequence) and the selective pressure on misfolding is very weak. We then increased this selective pressure by setting 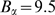 instead of 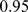. The resulting curve can be seen in Fig. 7 as a dashed curve. One finds that the maximum GC usage is now much larger, reaching 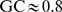.

**Figure 8:**
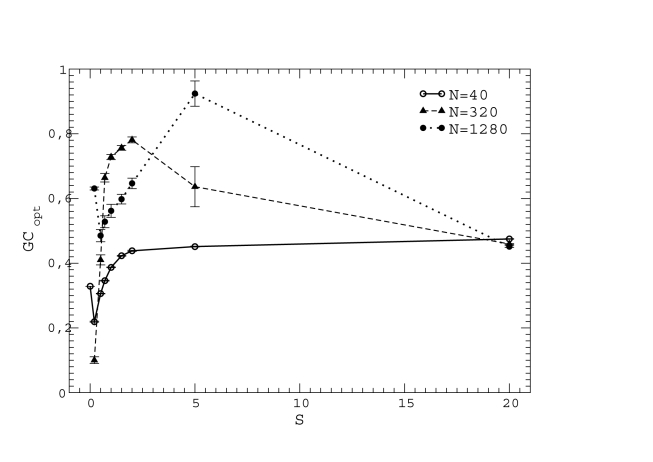
Optimal GC usage 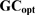 versus neutrality exponent 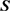 for three population sizes 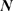.

Finally, we show in Fig. 8 the optimal GC usage versus the neutrality exponent 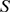 for small (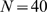), intermediate (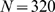) and large (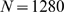) populations. For small populations the optimal GC usage increases with the neutrality exponent, from very small values to 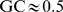. For intermediate and large populations the optimal GC usage has a maximum and then it decreases. The maximum value of 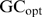 increases with population size, and it is reached at smaller neutrality exponent for intermediate populations (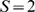 at 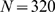) than for large populations (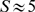 at 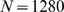).

We then tested the mean-field prediction that the stability coefficient 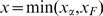 has a maximum and the sequence entropy has a minimum as a function of neutrality exponent 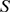. As expected, maximum stability and minimum entropy occur at the same value of 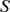, see Fig. 5 in the Text S1.

#### Qualitative behavior of the optimal GC

We now discuss the 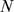-dependence of the optimal GC based on the results reported in Fig. 2. As explained above, the existence of the optimal GC usage arises from the trade-off between unfolding stability and misfolding stability in response to changes in the mutation bias. One can observe this trade-off in Fig. 2, from which it appears that 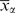 and 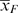 are negatively correlated for fixed population size. At the optimal GC the derivatives of 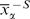 and 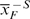 with respect to GC, which have opposite sign, become equal in absolute value, as indicated by Eq. (7). One can see from Fig. 2 that at small GC usage 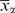 responds to GC variation more strongly than 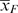, whereas the opposite happens at large GC usage, so that the optimal is reached at intermediate GC. In Fig. 2, the white thick line represents the 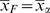 line at which the selective pressures on unfolding and misfolding are equal. One can see from the plot that, for small GC usage and small population sizes, the selective pressure is stronger on 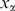 (misfolding). Since 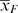 increases faster than 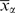 with population size, the selective pressure on 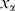 increases with 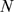 more than the selective pressure on 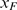. Consequently, the GC usage at which 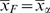 (white line) increases with population size. As discussed in the section “Influence of the mutation process”, this behaviour qualitatively explains why the optimal 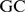 increases with 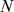 at small 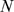, since the optimal 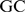 is expected to be near the value at which 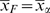. Near 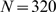, the optimal 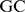 attains a maximum as a function of 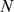. For 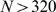, we see that 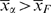 for all 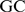 usages, so that the selective pressure is always stronger on misfolding, and we enter what we called the large 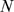 regime. In this regime, 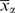 and 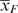 tend to the finite values that yield the maximum absolute fitness (numerical results suggest that they are 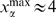 and 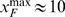), which are independent of GC, so that the GC dependence of stabilities gets weaker and weaker for large populations. When these limiting values are approached, the 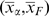 curves that correspond to fixed 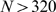 and varying 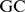 in Fig. 2 change their shape, becoming more convex and centered around 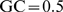 (red squares). This behavior corresponds to the fact that the optimal 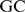 decreases towards 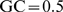 for very large population size.

According to this reasoning, the maximum value of 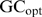 versus 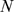 is reached at a population size where 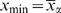 approaches its limiting value 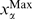. As discussed above and detailed in the Text S1, 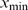 has a maximum as a function of 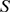 for fixed population size. Therefore, the population size at which a given value 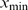 is reached has a minimum as a function of 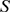, which implies that the population size 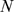 at which the optimal 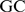 is largest has a minimum as a function of 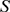. This prediction is in qualitative agreement with Fig. 4, bottom plot, which suggests that the minimum of the largest 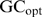 versus 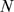, 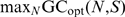, is reached between 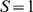 and 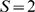.

### Effective population Size

The results that we have presented suggest that mutation bias towards AT or GC favor protein folding stability for very small and intermediate population sizes, respectively, while very large populations are advantaged in the absence of bias (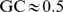). As it will be discussed below, this suggests that species evolving with mutation bias, either towards AT or GC, will have smaller population size than species with no bias. This prediction is consistent with the fact that almost all bacterial species with intracellular lifestyles, implying a reduction of effective population size through bottlenecks, shifted their mutation spectrum to AT, which resulted in small genomic GC content. On the other hand, among bacteria with large GC content some are facultative pathogens, such as *Mycobacterium tuberculosis*, and some live symbiotically in plant nodules, but there is no general tendency allowing for the deduction of their population size from their lifestyles. Therefore, to test our prediction, we tried to directly estimate their effective population size.

**Figure 9:**
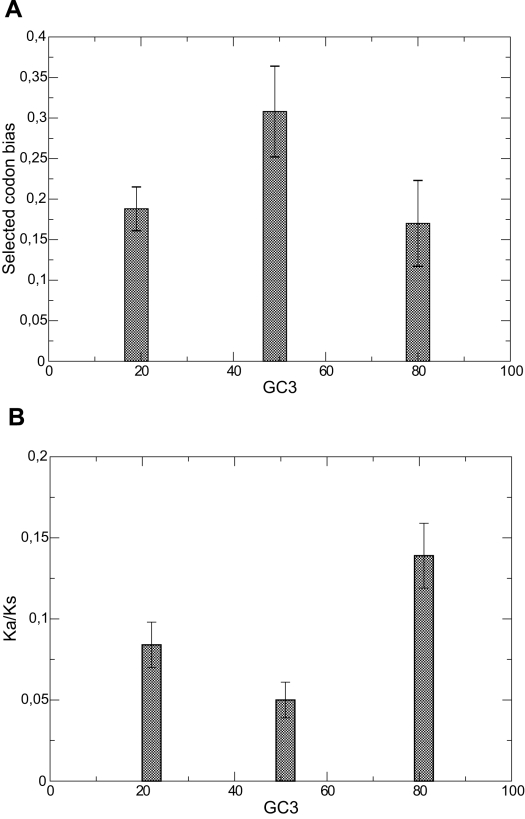
Estimates of quantities correlating with effective population size obtained from genomic data. Upper plot: Optimal codon bias estimated by dos Reis *et al.* [44] versus GC content at synonymous third codon position, shown as mean and standard error of the mean for three bins of GC3 (smaller than 30%, 40 to 60%, larger than 70%). Error bars in the plot represent the standard error of the mean, and show that the mean values are significantly different. However, data prior to the mean are rather broadly distributed, with standard deviations equal to 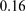 (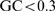, 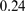 (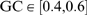) and 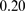 (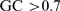). Bottom plot: values of 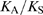 computed by Daubin and Moran [46] are averaged for pairs of bacteria with low, intermediate and high GC content. Both plots support the notion that species with GC content 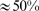 are characterized by larger effective population size.

The effective population size 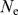 depends on the breeding structure and the natural history of a population, and in particular it is influenced by the bottlenecks that the population may undergo if a few individuals periodically colonize new environments. Therefore, the effective population size cannot be measured experimentally, but is estimated by fitting some observed population feature to its expected value under evolution in a population with given 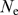. Optimal codon usage was used several years ago to estimate the effective population size of *Escherichia coli* [42]. A recent work supports the existence of a correlation between effective population size and synonymous codon usage [43], and the availability of many complete genomes makes it possible to analyze codon usage on a large scale. Codon usage and mutation bias are intimately correlated. It is commonly believed that the mutation bias, rather than selection for optimal codon usage, ultimately influences the global GC content of a genome [18], [19]. The definition of the optimal codon usage on which the results that we use here are based considers the excess frequency of preferred codons with respect to the frequency expected under mutation alone, and is therefore not expected to depend on the mutation bias in a trivial way. Dos Reis *el al.* [44] have recently estimated the optimal codon usage in a large number of prokaryotic species. We use their data rather than the analogous data obtained by Sharp *et al.* [45], since Dos Reis *et al.* evaluated the optimal codon usage on the entire genome, whereas Sharp *et al.* concentrated their attention only on ribosomal genes, which can be a biased sample. Fig. 9 shows the average optimal codon usage versus the average GC content at the third codon position, which is not affected by the selection on the amino acid sequence and is expected to be very strongly correlated with the mutation bias. We distinguished species with small (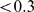), intermediate (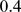 to 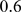) and large (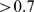) GC content. Species with intermediate GC content turned out to have significantly larger optimal codon usage, which suggests that they have larger effective population size. The scatter plot and the histogram of the GC content are shown in Fig. 7 and 8) in the Text S1. Error bars in the plot represent the standard error of the mean, and show that the mean values are significantly different. However, data prior to the mean are rather broadly distributed, with standard deviations equal to 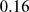 (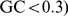, 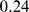 (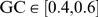) and 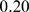 (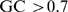).

As a second estimate of effective population size, we considered the ratio between non-synonymous and synonymous substitutions 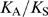, which is thought to represent the strength of negative selection [8]. We examined values of 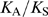 computed for pairs of entire genomes, recently published by Daubin and Moran [46]. From their table, we eliminated two pairs of genomes for which the evolutionary divergence, estimated through 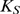, was very small (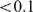), corresponding to *Bordetella pertussis/parapertussis* and two strains of *Xylella fastidiosa*, since it is known that the amino acid substitution rate is significantly higher at small time separation [47]–[49] and in fact these two pairs of genomes showed the two largest values of 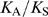. We also eliminated two pairs for which the two compared species had genomic GC content in different bins: two strains of *Prochlorococcus marinus* having GC = 36% and 51%, and the pair *Synechocystis/Synechococcus* having GC = 48% and GC = 65%, respectively. We divided the remaining 19 pairs in 3 bins of low, mean and high GC content and averaged their 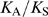. Results, shown in Fig. 9, clearly show that species evolving with no bias are characterized by lower 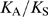, hence larger effective population size, in agreement with the analysis of the optimal codon usage and with the prediction of our model.

**Figure 10:**
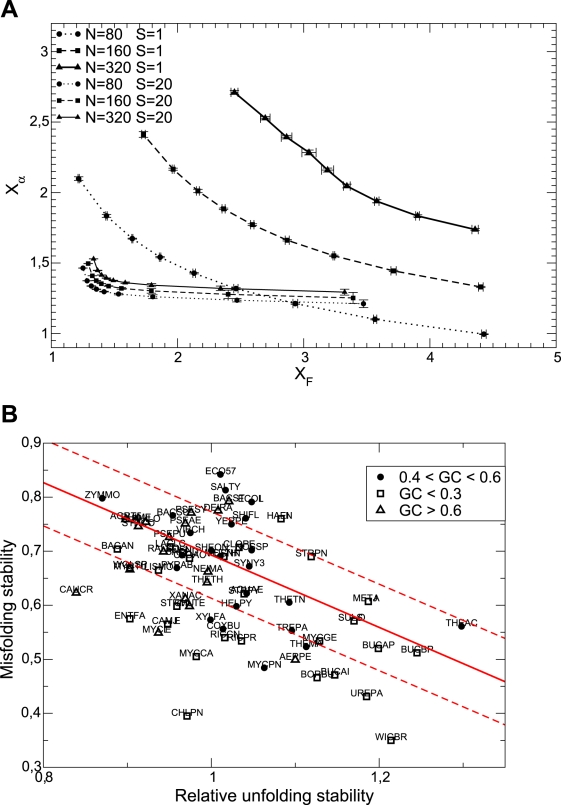
Negative correlation between misfolding and unfolding stability. Upper plot: Simulation results for average misfolding stability 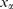 versus unfolding stability 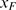 for various mutation biases, three population sizes and neutrality exponent 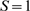 (non-neutral regime) and 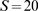 (neutral regime). Bottom plot: Estimated misfolding versus unfolding stability for families of homologous proteins in prokaryotic genomes (data from Ref. [12]). We distinguish genomes according to 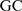 content at third codon position. The solid line represents a linear fit of misfolding stability for genomes with moderate or no mutation bias (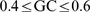).

**Figure 11:**
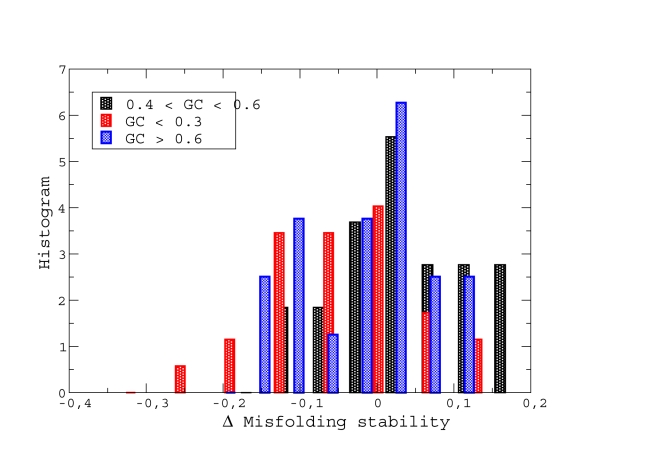
Relationship between GC usage and protein folding stability in orthologous proteins in different prokaryotic genomes (data taken from Ref. [12]). Histogram of the difference between the actual misfolding stability and the misfolding stability expected from the unfolding stability, using the relationship derived from species with moderate bias (continuous line in the previous plot). Notice that species with small and large GC usage have smaller than expected misfolding stability.

Finally, we reanalysed our data on protein folding stabilities computationally estimated for orthologous proteins in different prokaryotic genomes [12]. Unfolding and misfolding stabilities are negatively correlated, as predicted by our model (see Fig. 10). We found that most of the organisms evolving with mutation bias have proteins whose misfolding stability is lower than what could be expected based on their unfolding stability, see Fig. 11. This further supports the idea that these species are characterized by reduced effective population sizes.

## Discussion

### Interplay between mutation bias and population size

We studied here a mathematical model of protein evolution where the genotype to phenotype mapping is determined by the stability of the mutated protein against unfolding and misfolding, predicted using a protein folding model that correlates well with experimental measures. As observed in previous work, the two kinds of stability respond in an opposite way to changes in the GC usage of the mutation process. This fact produces a trade-off between the two kinds of stability, and an interesting phenomenology arises from the impossibility to find a mutation process that optimizes both stabilities at the same time, a concept that in the physical literature has received the name of *frustration*.

We considered three key evolutionary parameters: the effective population size 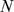, the neutrality exponent 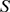, which determines how protein stability influences fitness, and the GC usage that expresses the mutation bias. Despite its importance in shaping the folding properties of proteins, the latter has been rarely considered in evolutionary models. Here we show that, in the non-neutral regime, mutation bias has a very interesting interplay with population size. We suggest that this can explain why some microbial species adopted extreme mutation bias.

At high neutrality exponent, all proteins with stability above the neutral threshold provide the same fitness and evolution is only able to attain the lowest allowed stabilities [3], almost independent of population size. Consistently, our analytic and numerical results indicate that the neutrality exponent 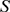 has a non-monotonic influence on protein stability, which reaches a maximum at intermediate 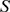 for given population size. The increase of 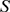 in our model has its biological counterpart in the increase of the expression level of chaperones, which make proteins more tolerant to stability losses. Therefore, the decrease of stability for increasing 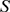 predicted by our model would correspond in the real world to the decrease of protein stability when the chaperone expression is increased. This outcome appears rather plausible. However, given the cost of synthesizing chaperones, in real evolution it is to be expected that the increase of the expression level of chaperones is a consequence of the loss of protein stability, as observed in intracellular bacteria with reduced population size, rather than the other way round.

In the neutral regime the GC usage influences the amino acid composition and consequently the folding properties, favoring proteins more stable with respect to misfolding but less stable with respect to unfolding, without modifying the fitness. In contrast, in the non-neutral regime fitness is a continuous function of stability and the outcome of evolution depends non-trivially on mutation in the sense that for fixed population size there is an optimal mutation bias at which fitness and stability are maximal. This is an unexpected result, which implies that mutation and selection are effectively entangled, and that the mutation spectrum constrains the maximum stability and fitness that an evolving population can attain. The possibility that the mutation rate is optimized as a response to evolutionary forces [50] has received considerable attention in experiments (see Ref. [51] for a recent work) and modelling (see for instance Refs. [52], [53]). The main forces influencing mutation rate evolution have been identified as the population size [50], the ruggedness of the fitness landscape [54] and the average negative effect of a mutation [55]. Recently, a theoretical work has established a relation between mutation rate, maximal genome size and thermodynamic response of proteins to point mutations, showing that populations go extinct via lethal mutagenesis when their mutation rate exceeds a few mutations per genome per replication [56]. Simulations of this model confirmed the predicted behaviour, showing that the limiting number of mutations is approximately seven for RNA viruses and about four for DNA-based organisms, with some weak dependence on the number of genes in the organism and the organism's natural death rate [57]. This model predicts that species with high mutation rates tend to have less stable proteins compared to species with low mutation rates. Therefore, the notion that the mutation process can influence protein stability, and that the optimal mutation process is influenced by properties of the selection process is not new, but the extension of this concept to the evolution of the mutation bias is novel to our knowledge.

Quite interestingly, small populations attain higher fitness with AT bias, intermediate populations get an advantage with GC usage, and very large populations attain higher fitness with almost absent bias. This result establishes a deep interplay between population size and mutation bias. The ML equations show that the optimal GC usage depends on how the number of stable sequences decreases with the stability values, i.e. it is an effect of probability in sequence space. For very small population size and stabilities the optimal mutation bias is attained at small GC usage, which makes folding easier. At higher stabilities (intermediate population size) the optimal GC usage increases, therewith improving the stability against misfolding at the optimal GC. Approaching the maximal stabilities the optimal GC usage decreases again towards the value 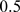, which means absence of bias in the mutation process.

As a speculative remark, we note that it was not obvious that our model would predict 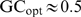 as the optimal GC usage for very large populations. In this limit the absolute maximum fitness is reached. We have shown numerically (see Text S1) that the optimal GC usage in the infinite population limit is little dependent on the parameters of the fitness function 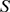, 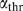 and 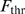, as long as the selective pressure affects mostly 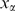, so that in this limit 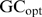 mainly depends on the contact energy parameters and on the genetic code. This conjecture is consistent with our data. Nevertheless, a systematic test requires cumbersome simulations that we did not perform here. We obtained a different result when using the GKS contact energy parameters, which yielded 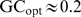 for 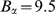 in the very large population limit. However, we notice that these parameters also produced a very small normalized energy gap, which suggests that they might be less suitable for this kind of study.

### Influence of the mutation rate

The model that we adopt here is based on the assumption that the population is genetically homogeneous, i.e. the product 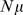 of population size times mutation rate is small. This allows us to analytically compute the fixation probability of a new mutation through Eq. (1) instead of explicitly simulating population dynamics. This approximation is considered valid if 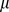 measures the mutation rate of a single protein, in particular if population size is small. However, the high mutation rates of RNA viruses may violate this assumption even for a single protein, and in this case several works [58], [59] have shown that the load due to nonviable mutations significantly modifies the evolutionary process even in the case of a neutral fitness landscape, leading to the evolution of mutational robustness and enhanced folding stability [60]–[62]. This situation can be studied analytically in the framework of the quasi-species theory [63]. We did not consider this theory here, because it assumes that the population size is infinite and therefore it prevents to study the effect of finite populations that is the main focus of the present work. If we considered a whole evolving genome instead of a single protein, the approximation of very small mutation rate would not be justified, since genomic mutation rates are in a range of 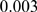 to 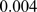 mutations per genome per generation for DNA-based microbes, including viruses, bacteria, and eukaryotes [55]. In this context, a new interesting effect has to be considered, namely the hitch-hiking effect, which consists in the fixation of mildly disfavoured alleles driven by a positively selected allele present in the same chromosome. However, since treating the hitch-hiking effect would make both the analytic and the numeric study much more complicated, we leave it as a subsequent step.

### Robustness of the results

Our model depends on several assumptions and parameters. As evolutionary model, we adopted the Moran process, one of the best studied population genetic models. The theoretical work by Sella and Hirsh [17] shows that other evolutionary processes, such as for instance the Wright-Fisher process, would yield the same qualitative results. The mutation process was modelled using a single parameter, the GC usage. While this parametrization might appear too simplified, it has the merit to focus on a variable whose relevance has been pointed out by a large number of experimental studies, statistical analysis and models.

The ingredients of our model that seem more debatable are the form of the fitness function and its parameters 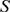, 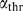 and 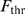. To test the robustness of our results, we simulated different functional forms of the fitness function, using exponential functions of stability instead of power laws or letting the fitness depend only on the minimum between the two stabilities 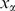 and 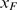. In all cases, we found the same qualitative results: There is an optimal mutation bias at which the fitness is maximal, such that for very small populations the optimal bias is towards AT, and for intermediate populations the optimal bias is towards GC. We then studied in detail the fitness function Eq. (2). Changing the neutrality exponent does not modify the qualitative results as long as the combination of 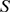 and 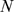 is in the non-neutral regime. Experiments on the evolution of small populations [13], [14] and computational studies of protein folding stability [12] suggest that stability does depend on population size for populations subject to repeated bottlenecks, so that for such populations it is justified to assume that the non-neutral regime is the relevant evolutionary regime. We also varied the neutral thresholds 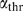 and 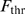 by more than 20%, finding that they do not change the qualitative behavior, although they have a quantitative influence on the optimal GC usage. We observed more important quantitative changes when we changed the contact energy parameters, but even in this case the gross qualitative features of the 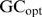 versus 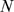 relationship remain valid.

### Meta-population evolution of the optimal bias

The result that the mutation bias directly influences the fitness that a population can attain in its evolution suggests the intriguing possibility that there may be a feedback between mutation and selection such that a particular mutation bias favors optimal protein folding stability, and selection may favor the replication machinery yielding this optimal mutation bias. Nevertheless, the selective advantage of evolving with the optimal GC usage is only apparent after a sufficiently large number of substitutions in protein coding genes. A mutant for GC usage would have a very low selective advantage during the first generations, and therefore its fixation would be a matter of almost neutral genetic drift. After the mutant is fixed, however, our model predicts that the population evolving with optimal bias will accumulate a sufficiently high selective advantage to take over populations with a less favourable GC usage when they, or their hosts in the important case of endosymbiotic bacteria, come to compete. Therefore, we expect this meta-population selection to almost deterministically favour the selection of the strain with optimal GC usage in contrast to the almost neutral fixation of a mutant with optimal GC usage within a single population. Thus the optimal mutation bias can facilitate the selection of more stable proteins and, on a longer time scale, selection at the meta-population level may favor the replication machinery that is most suitable to protein stability.

The population sizes at which we find the maximum of 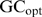 in our model are of the order of a few hundreds individuals for 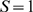. These values appear very small compared with real bacterial populations, even if they tend to grow rapidly for very high or very low neutrality exponent 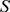. We may reconcile our model with biology if we notice that the effective population size is not the same as the total number of individuals of a species. Berg [42] showed that, if a small number of individuals often colonize new habitats with colonization probability almost independent of the founders fitness, the effective population size is given by the number of generations between two colonization events. This is a very small number for obligatory endosymbiotic and parasitic bacteria, and it may also be small for facultative parasites or symbionts, and even for the paradigm of a free living bacterium such as *Escherichia coli* for which Berg [42] estimated an effective population of 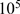 individuals.

The meta-population structure of bacterial species raises the question of whether the molecular evolution properties of a species such as the codon usage bias and the 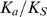 ratio are primarily determined by the effective size of a local population or by the global size of the meta-population. This is an important question that requires modelling the meta-population dynamics and the different levels of selection that are relevant for it. Our opinion is that both population sizes influence the evolutionary dynamics, and that, despite the losses of stability of small local populations can be in part compensated at the meta-population level, the influence on evolution of the local population size remains important even taking into account these corrections, so that observables such as codon usage bias and 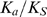 strongly reflect the local structure of the population.

### Comparison with observed mutation bias

The distribution of GC content observed in bacterial genomes is remarkably broad. We assume here, as it is widely believed, that these differences in the GC content are mainly determined by different mutation pressures [18], [19]. The third codon position, where a shift from A to G and from C to T does not change the coded amino acid in most cases, is thought to strongly reflect the mutation bias. However, the GC content at third codon position is strongly correlated with the GC content at first and second codon position [20], [21], and through this correlation, the mutation bias influences the properties of the protein sequence, most notably its hydrophobicity [12], [22]. This is surprising, since hydrophobicity is considered the main determinant of folding stability [23], and it is expected to be finely tuned since the protein has to avoid unfolding on one hand, and misfolding and aggregation on the other hand (of course this balance is very different for membrane proteins, which are not considered here). One possible interpretation is that, due to the trade-off between unfolding and misfolding, the hydrophobicity is to some extent neutral so that it is possible to modify it without significantly affecting the global fitness of the protein. Our results suggest a different interpretation: There may be an optimal range of hydrophobicity, but this range may be different for different values of protein stability. So proteins with low stability, as those found in small populations, may tend to be more hydrophobic than proteins with high stability as those found in large populations, hence leading to a preference for a lower GC usage in their evolution.

Our model predicts that species with large population size will tend to evolve without mutation bias (GC usage equal to 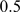), whereas species with small and intermediate populations will tend to present such a bias, either towards AT or towards GC. This prediction is in qualitative agreement with two independent estimates of effective population size based on optimal codon usage and on the ratio between non-synonymous and synonymous substitutions represented in Fig. 9, and with a computational comparison of unfolding and misfolding stabilities in orthologous bacterial proteins, see Fig. 11. Of course bacterial genomes are rather complex, and we do not expect the mechanism proposed here to explain their GC content as the result of a single factor, population size. Another important factor influencing the GC content has been identified in a previous statistical study, which demonstrated that aerobiosis is an important determinant of GC rich genomes [64]. This interesting result is not in contradiction with our model, since many bacteria with small GC content tend to have an intracellular lifestyle, which in turn can make them anaerobic and at the same time reduce their effective population size.

As mentioned above, the proposed relationship between low GC content and small population size is consistent with the known fact that most bacterial species that adopted an intracellular lifestyle shifted their mutation spectrum towards AT with respect to their free living relatives [26]. This AT bias is, in most cases, the consequence of the loss of repair genes. For instance, three out of the four sequenced species of *Buchnera* lost the gene mutH, which in *Escherichia coli* is responsible of repairing the replication errors produced by methylation of cytosine that causes C to T mutation [65]. Moran proposed that this loss of repair genes and the consequent mutation bias is a selectively nearly neutral event in the evolution of endosymbionts [9]. Nevertheless, the results presented here suggest that this shift has important consequences on the folding properties of the whole proteome. In fact, a strong AT bias, together with reduced population size, is expected to produce severe misfolding problems, as indicated by the low predicted misfolding stability of proteins of intracellular bacteria with respect to orthologous ones in free living bacteria [12], and by the observed positive selection and over-expression of molecular chaperones in endosymbiotic bacteria [66], which is an expensive but effective strategy to reduce misfolding problems. Interestingly, it has been found that the fitness observed in an experimental population subject to frequent bottlenecks can be in part recovered by over-expressing chaperones [15]. Nevertheless, AT bias also enhances stability with respect to unfolding, and the results presented here suggest that its influence on fitness is globally positive for small populations.

The relationship between small population size and GC richness is even less expected. Only a few out of several prokaryotic species having high GC content are obligatory intracellular bacteria, such as for instance *Mycobacterium leprae*, and some are facultative pathogens or plants associated symbionts. Our results suggest the intriguing possibility that they tend to have small population size, although larger than for obligatory endosymbionts. To test this prediction, we estimated the population size using optimal codon usage [44], which has often been used to estimate population sizes. There are several caveats: The selective advantage of optimal codon usage strongly varies from one gene to another, and from one species to another. However, it is expected that the average codon usage bias estimated on the whole genome is correlated with population size. The optimal codon usage is computed subtracting the average mutation background, therefore it should not be trivially influenced by mutation bias. We found significantly reduced selection for optimal codon usage in bacteria evolving with large mutation bias compared to those with moderate or no bias, supporting our prediction that the former are characterized by smaller effective population size. Furthermore, we tested the relationship between GC content and effective population size estimating the latter through the ratio between non-synonymous to synonymous substitutions computed by Daubin and Moran [46] for entire bacterial genomes. This analysis presents important caveats. For instance, the non-synonymous substitution rate has been shown to depend on the time separation between two species [47]–[49]. We tackled this point by eliminating values of 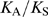 estimated at short timescales, which are known to be strongly overestimated. Given the above, it is remarkable that the qualitative picture provided by this measure qualitatively coincides with the one obtained analysing optimal codon usage. Both measures strongly support the prediction of our model that species with 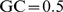 are characterized by larger effective population size. Nevertheless, among species presenting large mutation bias, those with bias towards GC are estimated through the 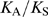 measure to have smaller effective population than those with bias towards AT, which is in contrast with our prediction. This point is worth further investigation taking into account more carefully the time dependency of the 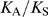 estimate [48].

Of course, there exist several exceptions to these predictions, as there are several other factors, some already identified [64], [67] and others still unknown, that influence the differences in GC content of prokaryotic species. One remarkable exception to the association between intracellularity and low GC content is the genome of the endosymbiotic bacterium *Hodgkinia cicadicola*, very recently sequenced by Moran's group [68]. This genome is extremely reduced (144 kb), as generally observed for endosymbiotic bacteria, but it shows GC content of 58%, which came as a big surprise since it is probably the most serious exception to the association between genome size and GC content. This genome also challenges the association between endosymbiotic bacteria and AT bias. It has been suggested that *Hodgkinia* belongs to the Rhizobiales division of alpha proteobacteria, characterized by high GC content. Interestingly, the genetic code of *Hodgkinia* underwent a modification such that UGA codes for Tryptophan instead of Stop. This modification is expected to ease the evolution of proteins that are stable with respect to misfolding. Consistently with this expectation, we found that the optimal GC usage for small populations slightly increases when this alternative genetic code is used in simulations, but this effect is too small to reconcile the GC content of *Hodgkinia* with its expected small effective population size (data not shown). Further research is needed to identify the origin of the GC content in this genome that lacks any repair gene [68]. Nevertheless, the association between intracellular lifestyle and AT bias, despite not being deterministic as demonstrated by this counterexample, is still strongly significant.

A second exception is represented by *Prochlorococcus marinus*, a very abundant species of small marine cyanobacteria [69], [70]. It is expected that this species has a very large population size, which is in agreement with a recent estimate of its 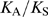 ratio [46]. 11 out of 13 fully sequenced strains of this cyanobacterium present low GC content, in the range between 30 to 38 percent, apparently contradicting the association between large population size and lack of mutation bias. However, the two remaining strains have GC content of 50%, as expected according to our model, and one of these was used to estimate the small 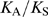 ratio that supports the large population size. *Prochlorococcus* has a complex meta-population structure in which the strains with 50% GC content, characterized by large genomes, appear to act as gene reservoirs. These strains are also characterized by a larger cell size than other *Prochlorococcus* strains, which the authors describe as “a feature that may have led to their lower isolation recovery due to the filtration step most often used to separate *Prochlorococcus* from *Synechococcus*. Hence, there are probably more LL-adapted *Prochlorococcus* strains with cell and genome sizes similar to those of *Synechococcus* thriving deep in the euphotic zone. This is apparently confirmed by the dominance of this ecotype at the base of the euphotic zone in the Atlantic Ocean, as revealed by quantitative PCR data” [70]. These strains with large genomes and without mutation bias are found at considerable depth in the ocean and thus at low oxygen pressure. There seems to be a positive association between ocean depth and GC content for *Prochlorococcus* strains, thus a negative association between oxygen pressure and GC content, opposite to the observed general association between oxygen and GC content [64]. Comparative analysis of the sequenced *Prochlorococcus* strains will be necessary to test the hypothesis that there is an association between the GC content and the population size of these strains. Consistent with this possible association, it was found that in the MED4 strain, characterized by the smallest GC content among all *Prochlorococcus* strains, translational selection does not shape the codon usage variation among the genes in this organism [71].

### Conclusions

We have shown here that the AT mutation bias can increase the fitness associated with essential proteins if the population size is very small. The same happens with GC mutation bias for intermediate population. These results suggest that the mutation bias is not selectively neutral, but it may be the preferred outcome for the evolution of small populations. We found a deep interplay between the estimated effective population size and the GC content that is consistent with the predictions of our model. Of course this association is not deterministic, since many other factors influence the GC content. However, the influence of population size is an intriguing one that we believe is worth further investigation. Thus, we hope that this proposal will be subject to experimental test in the future.

## Materials and Methods

### Folding stability

As in our previous work, the unfolding free energy of a protein with sequence 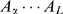 and contact matrix 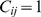 if the minimal interatomic distance between residues 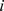 and 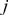 is below 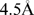, 0 otherwise, is defined as
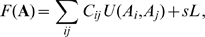
(9)where 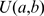 is the contact interaction matrix determined in [72], 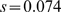 was determined fitting Eq. (9) to a set of experimentally measured unfolding free energy (UB, unpublished) and 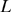 is protein length. Although rather simple, this model is accurate enough to allow quantitative predictions of the folding free energy of small proteins that fold with two-state thermodynamics (the correlation coefficient between experimental and predicted free energy is 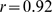 over a representative test set of 20 proteins, UB, unpublished result) and of the stability effect of mutations (correlation coefficient 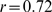 over a set of 195 mutations, UB, unpublished result). This is comparable to state-of-the-art programs such as Fold-X [73]. However, the computational simplicity of the model makes it affordable to use it for simulating very long evolutionary trajectories with a large number of parameters, which would not be possible using other tools.

The normalized energy gap 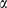 measures how alternative compact conformations are higher in energy than the native, and it is defined using the random energy model [74], [75] as
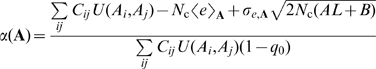
(10)with 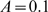, 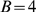, 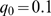, 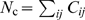, and 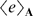 and 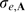 are the mean and standard deviation of the interaction energy of both native and non-native contacts in sequence 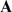.

### Protein structures

We studied five proteins with different size and secondary structures: Phosphocarrier protein of *E.Coli* (85 amino acids, PDB id. 1opd), Lysozyme of *G.Gallus* (129 amino acids, PDB id. 3lzt), ATP synthase epsilon chain of *E.Coli* (135 amino acids, PDB id. 1aqt), Triose Phosphate Isomerase of *E.Coli* (255 amino acids, PDB id. 1tre) and Tryptophan Synthase alpha chain of *S. Typhimurium* (260 amino acids, PDB id. 1a50). When not otherwise stated, we exemplify our results with the structure of the protein lysozyme.

### Mutation process

Mutations are modelled through the HKY process [28], in which the mutation rate from nucleotide 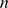 to 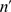, 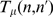, is 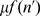 if 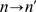 is a transition, 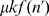 if it is a transversion. The transition/transversion ratio is fixed at 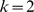. The microscopic rate 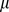 is assumed to be very small and it does not affect the results. We further assume 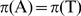 and 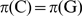 (Chargaff second parity rule), so that the only parameter of the mutation model is the stationary GC content, 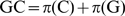, which we call GC usage.

### Simulation of the evolutionary process

Simulations were performed starting from the native sequence, which was changed through random mutations subject to the acceptance probability Eq. (1) computed using the estimated folding stabilities. We checked that simulations converged in all cases after a number of accepted substitutions not larger than a few times the protein length 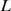, and discarded the first 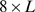 steps of the trajectory for collecting statistics. The simulations were run until 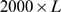 accepted substitutions were collected, which makes it rather cumbersome to simulate large populations for which the acceptance rate is small. For each set of parameters we run 10 independent simulations in order to evaluate the statistical error.

At every step, we randomly draw one mutating DNA site 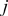 with probability dependent on the nucleotide 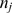 that occupies it, 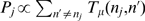, and we draw a new nucleotide 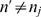 with probability proportional to 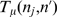. The mutation is then translated to the amino acid sequence, whose stability is computed through Eq. (9) and (10) from which we obtain fitness through Eq. (2). The fitness is compared to the one of the current wild type sequence and the mutation is accepted with probability given by Eq. (1).

### Optimal mutation bias

For fixed 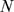 and 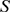 the equilibrium fitness 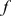 is simulated for 9 GC usages from 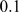 to 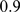 and the results are fitted to a cubic function, from which we obtain the optimal 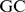 at the point where the first derivative vanishes. If 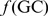 is monotonically increasing or decreasing the maximum (minimum) 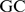 is chosen. To estimate the error, we estimated 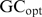 from 10 independent simulations, and we computed mean and standard error of the mean.

## Supporting Information

Text S1 Supporting figures and analytic developments
(0.23 MB PDF)
