# Supplementary material for: Mutation Bias Favors Protein Folding Stability in the Evolution of Small Populations
Source: PLoS Comput Biol. 2010 May 6;6(5):e1000767. doi: 10.1371/journal.pcbi.1000767 (PMC2865504; doi:10.1371/journal.pcbi.1000767)
Supplement: Text S1 — Supporting figures and analytic developments (0.23 MB PDF) [file pcbi.1000767.s001.pdf]

Supporting Information for
Mutation bias favors protein folding stability in the evolution of small populations

Raul Mendez*,[1] Miriam Fritsche*,[2,†] Markus Porto,[2,‡] and Ugo Bastolla[1,§]

[1]*Centro de Biología Molecular "Severo Ochoa", (CSIC-UAM), Cantoblanco, 28049 Madrid, Spain*
[2]*Institut für Festkörperphysik, Technische Universität Darmstadt, Hochschulstr. 8, 64289 Darmstadt, Germany*

Content

1. **Analytic calculations**
2. **Supporting figures**
   - **Fig. 1:** Phase diagram
   - **Fig. 2:** Approach to the stationary distribution of fitness
   - **Fig. 3:** Large population limit of the optimal mutation bias
   - **Fig. 4:** Difference in selective coefficients versus population size
   - **Fig. 5:** Stability and reduction in sequence entropy versus S
   - **Fig. 6:** Mutation bias dependence of $|\partial\sigma/\partial x|_{x=1}$ in the neutral limit.
   - **Fig. 7:** Scatter plot of the optimal codon usage (estimating the effective population size) versus GC content at third codon position for prokaryotic species
   - **Fig. 8:** Histogram of the GC content from the previous figure.

---

* These authors contributed equally to this work.
[†]Present address: Institut für Theoretische Physik, Ruprecht-Karls-Universität Heidelberg, Philosophenweg 19, 69120 Heidelberg, Germany
[‡]Corresponding author, email porto@thp.uni-koeln.de; Present address: Institut für Theoretische Physik, Universität zu Köln, Zülpicher Str. 77, 50937 Köln, Germany
[§]Corresponding author, email ubastolla@cbm.uam.es

Analytic calculations

A. Maximum likelihood equations

We can analytically predict how the population size N and the neutrality parameter S influence stability and fitness by exploiting the formal analogy between population dynamics and statistical mechanics proposed by Sella and Hirsh [1]. They noticed that, for monomorphic populations for which fixation events only involve the wild-type genotype and a single mutant, several evolutionary processes studied in population genetics tend to a stationary fitness distribution of the form $\exp(N\varphi)$ that is formally equivalent to a Boltzmann distribution, with population size N playing the role of inverse temperature and the logarithm of fitness $\varphi = \log(f)$ playing the role of minus energy. This selective process has to be combined with the mutation process. We define $P_{\rm mut}(\alpha, F, \mathrm{GC})$ as the probability to find stability values α and F under an evolutionary process with no selection (f is constant for all genotypes) and a mutation process with given GC usage (i.e., the stationary GC content of the evolutionary process is GC). Combining mutation and selection, we can compute the probability to observe misfolding and unfolding stability α and F in the stationary state of a population of N evolving individuals as

$$P_{\rm sel}(\alpha,F) \propto P_{\rm mut}(\alpha,F)\exp\left[N\log f(\alpha,F)\right] = \exp\left[\sigma(\alpha,F,\mathrm{GC}) + N\varphi(\alpha/\alpha_{\rm thr}, F/F_{\rm thr}, S)\right], \tag{1}$$

where we introduced the notation $\varphi = \log(f)$ and $\sigma(\alpha,F,\mathrm{GC}) = \log\left(P_{\rm mut}(\alpha,F,\mathrm{GC})\right)$. This latter quantity can be interpreted as the entropy in sequence space compatible with stabilities α and F in the absence of selection, which depends on the mutation process, hence on the GC usage. We also define the normalized stabilities $x_\alpha = \alpha/\alpha_{\rm thr}$ and $x_F = F/F_{\rm thr}$.

Since σ is proportional to sequence length L, and both L and N are large in biologically relevant situations, the distribution described by Eq. (1) is narrowly peaked around the values $\overline{x}_\alpha(S,N,\mathrm{GC})$ and $\overline{x}_F(S,N,\mathrm{GC})$ that have maximum probability, i.e. the stabilities that maximize the "evolutionary free energy" $G = \log(P_{\rm sel}) \equiv \sigma + N\varphi$. In the following, we take a mean-field perspective and we identify the mean stabilities with the maximum likelihood stabilities $\overline{x}_\alpha(S,N,\mathrm{GC})$ and $\overline{x}_F(S,N,\mathrm{GC})$, and the mean fitness with the fitness corresponding to these values, $\langle\varphi(x_\alpha,x_F,S)\rangle \approx \varphi(\overline{x}_\alpha,\overline{x}_F,S)$. We consider the additive fitness function (see main text)

$$\varphi(x_\alpha,x_F) \equiv \log(f) = -\log\left(1 + x_\alpha^{-S} + x_F^{-S}\right) \tag{2}$$

for positive x_α and x_F, and $\varphi = -\infty$ if either stability is not positive. The fitness takes the value $f = \exp(\varphi)$ if both stabilities are positive and $f = 0$ if either x_α or x_F are negative, which are strictly forbidden. Fitness grows with stability. The maximum value of stability is of course finite, since the number of sequences is finite. Fitness becomes a binary value $f \in \{0,1\}$ when S tends to infinite, with $f = 1$ when stabilities are above the neutral thresholds $x_i = 1$ and $f = 0$ when either stability is below the threshold. We call this the neutral limit.

With now consider the logarithm of the probability in Eq. (1),

$$G(x_\alpha,x_F,N,S,\mathrm{GC}) \equiv \sigma(x_\alpha,x_F,\mathrm{GC}) + N\varphi(x_\alpha,x_F,S) \equiv \sigma(x_\alpha,x_F,\mathrm{GC}) - N\log\left(1 + x_\alpha^{-S} + x_F^{-S}\right). \tag{3}$$

The quantity $-G$ can be the interpreted as an evolutionary free energy. The most likely stability values $\overline{x}_\alpha$ and $\overline{x}_F$ maximize G or, equivalently, they minimize the evolutionary free energy. They can be computed solving the maximum likelihood (ML) equations

$$\left[\frac{\partial\sigma}{\partial x_i}\right]_{x_i=\overline{x}_i} = -N\left[\frac{\partial\varphi}{\partial x_i}\right]_{x_i=\overline{x}_i} = -NS\frac{x_i^{-S-1}}{1 + x_\alpha^{-S} + x_F^{-S}}, \tag{4}$$

with $i = \alpha, F$. These equations express the balance between the relative decrease in the number of sequences with enhanced stability due to mutation entropy and their relative increae due to selective pressure. Validity of the ML equations requires that G has a maximum, so that the Hessian matrix H of the second derivatives of G must be negative definite. This matrix is defined by

$$H_{ij} \equiv \frac{\partial^2 G}{\partial x_i \partial x_j} = \frac{\partial^2\sigma}{\partial x_i \partial x_j} + N\frac{\partial^2\varphi}{\partial x_i \partial x_j}. \tag{5}$$

This is the sum of the Hessian of $\varphi(x_\alpha,x_F) = -\log(f)$, which is negative by construction, as it is easy to verify, and the Hessian of $\sigma(x_\alpha,x_F)$, which is the logarithm of the probability to find stability values x_α and x_F under mutation alone. We assume that $\sigma(x_\alpha,x_F)$ has a single maximum at $x_\alpha = x_\alpha^{\rm mut}$, $x_F = x_F^{\rm mut}$. Therefore, $\partial\sigma/\partial x_i$ are negative for $x_i > x_i^{\rm mut}$, as it is required for the existence of solutions of the ML equations, and the Hessian of σ is negative, as required. We can go beyond the ML approximation writing the exponent G at second order as $G(x_\alpha,x_F) \approx G(\overline{x}_\alpha,\overline{x}_F) + \frac{1}{2}\sum_{ij} H_{ij}(x_i - \overline{x}_i)(x_j - \overline{x}_j)$, which is equivalent to approximating the distribution $P_{\rm sel}$ as a Gaussian with covariance matrix $-H^{-1}$. Therefore, negativity of the Hessian matrix is equivalent to requiring that the covariance matrix is positive.

The definition of the normalized energy gap α implies that $x_\alpha^{\rm mut}$ is always negative. In contrast, our numerical results show that $x_F^{\rm mut}$ is positive for small GC usage corresponding to very hydrophobic sequences.

¿From the ML equations, we can obtain the following implicit equations that expresses the stability $\overline{x}_F$ as a afunction of $\overline{x}_\alpha$.

$$\overline{x}_F^{S+1}\frac{\partial\sigma}{\partial x_F}=\overline{x}_\alpha^{S+1}\frac{\partial\sigma}{\partial x_\alpha}\,. \tag{6}$$

We see from this equation that the smaller stability $\overline{x}_i$ is the one for which the absolute value of the derivative $\partial\sigma/\partial x_i$ is larger.

Influence of parameters on stability

We now calculate how $\overline{x}_\alpha$ and $\overline{x}_F$ depend on the parameters $\lambda\in\{N,S,{\rm GC}\}$. We perform this calculation by taking the total derivative of the ML equations $\partial G/\partial x_i=0$ with respect to the parameter λ and equating it to zero, since the ML equations must be satisfied for all values of λ. The total derivative is the sum of the partial derivative with respect to λ plus the partial derivatives with respect to $\overline{x}_j$ multiplied times $\partial\overline{x}_j/\partial\lambda$. We therefore get $\left(\partial^2G/\partial\lambda\partial x_i\right)+\sum_j\left(\partial^2G/\partial x_i\partial x_j\right)\left(\partial\overline{x}_j/\partial\lambda\right)$, from which we finally obtain

$$\frac{\partial\overline{x}_i}{\partial\lambda}=-\sum_j H_{ij}^{-1}\frac{\partial^2G}{\partial\lambda\partial x_j}\,, \tag{7}$$

where $H_{ij}=\left(\partial^2G/\partial x_i\partial x_j\right)$. As mentioned above, the inverse of the Hessian matrix can be interpreted as minus a covariance matrix and it is negative definite. For $\lambda=N$ we find

$$\frac{\partial\overline{x}_i}{\partial N}=-\sum_j H_{ij}^{-1}\frac{\partial\varphi}{\partial x_j}\,, \tag{8}$$

which is always positive, since $-H_{ij}^{-1}$ is a positive matrix and both $\partial\varphi/\partial x_j$ are positive. Therefore, both stabilities always increase with population size, consistent with the statistical mechanical analogy described above. For very small N, and provided that S is not too large so that SN is small, stabilities satisfy the equations $\overline{x}_i^{S+1}\left(\partial\sigma/\partial\overline{x}_i\right)\approx 0$, whose solution is either $\overline{x}_i\approx 0$ (for the case of x_α this is the only possible solution) or $\partial\sigma/\partial x_i\approx 0$, $\overline{x}_i\approx x_i^{\rm mut}$, which is only possible if $x_i^{\rm mut}$ is positive as it is the case for $x_F^{\rm mut}$ when the GC usage is small. For very large N stabilities approach the maximum possible values. However, they can not be maximized simultaneously since there is a trade-off between the two kinds of stability (see main text).

For $\lambda={\rm GC}$, we find

$$\frac{\partial\overline{x}_i}{\partial{\rm GC}}=-\sum_j H_{ij}^{-1}\frac{\partial^2\sigma}{\partial{\rm GC}\partial x_j}\,. \tag{9}$$

Numerical results show that $\overline{x}_\alpha$ is an increasing function and $\overline{x}_F$ is a decreasing function of GC usage, so that these two variables are anticorrelated when GC is varied.

Finally, for $\lambda=S$ we find

$$\frac{\partial\overline{x}_i}{\partial S}=-N\sum_j H_{ij}^{-1}\frac{\overline{x}_j^{-S-1}\left[1+\overline{x}_j^{-S}-S\log(\overline{x}_j)+\overline{x}_k^{-S}\left(1+S\log(\overline{x}_k/\overline{x}_j)\right)\right]}{\left(1+\overline{x}_\alpha^{-S}+\overline{x}_F^{-S}\right)^2}\,, \tag{10}$$

where k is the stability different from j (if $j=\alpha$ than $k=F$ and the other way round). The term in round brackets in the numerator can vanish at some value of S, meaning that stabilities need not to be monotonic function of S. For $S=0$ stabilities only have to fulfill the conditions $\overline{x}_i>0$, and their values are given by $\overline{x}_i=\min(x_i^{\rm mut},0)$. Our numerical results indicate that stabilitiies increase with S for small S. An N-dependent maximum is reached at intermediate S when both terms in round brackets with $j=\alpha$ and $j=F$ in Eq. (10) vanish. It is possible to see that the maximum can only be reached when both $\overline{x}_i$ are larger than one. For large S the fitness landscape is almost neutral, so that finite differences in stability determine very small differences in the additive fitness φ such that $N\Delta\varphi\ll 1$. Such differences can not be fixed in the population. In the limit $S\rightarrow\infty$ and for finite N, fitness only depends on the smaller of the two stabilities, $x=\min(x_\alpha,x_F)$, which tends to the value $x=1$, i.e. to the neutral threshold at which fitness approaches the value $f=1$. Therefore, stability is predicted to decrease with S at large S. This prediction is confirmed by numerical results. To simplify the analytic calculations, we also confirmed the prediction that stability has a maximum at intermediate S by using the simplified fitness function $\varphi=-\log(1+x^{-S})$, with $x=\min(x_\alpha,x_F)$ (see below).

Influence of parameters on fitness

Since fitness is an increasing function of stability and stability increases with population size, fitness always increases with population size, as expected. However, fitness is not a monotonic function of S but it starts from the value $f = 1/3$ at $S = 0$, it decreaes to a minimum at small S and then grows towards the neutral limit $f = 1$ at large S.

Optimal mutation bias

We now consider the dependence of the mutation bias on fitness. The two stabilities x_α and x_F depend on the mutation process through the sequence entropy $\sigma(x_\alpha, x_F, \mathrm{GC})$. High GC usage favors hydrophilic proteins, enhancing x_α at the expenses of x_F. Since fitness depends on both stabilities, there is an optimal mutation bias at which the fitness is maximal for given S and N, satisfying the ML equations (4) plus the implicit equation $\mathrm{d}\varphi/\mathrm{dGC} = 0$. Using the ML equations, we can write

$$\frac{\mathrm{d}\varphi}{\mathrm{dGC}} = \frac{\partial\varphi}{\partial x_\alpha}\frac{\partial \overline{x}_\alpha}{\partial \mathrm{GC}} + \frac{\partial\varphi}{\partial x_F}\frac{\partial \overline{x}_F}{\partial \mathrm{GC}} = 0\,. \tag{11}$$

The fitness is a decreasing function of $\delta = x_{\min}^{-S}\left[1+(x_{\min}/x_{\max})^S\right]$. In the neutral limit $S \to \infty$, $x_{\min}$ tends to 1 independent of GC usage and the fitness is maximal for $\overline{x}_\alpha = \overline{x}_F = 1$. From the saddle-point equations, this condition implies $\partial\sigma/\partial x_F(1,1,\mathrm{GC}) \approx \partial\sigma/\partial x_\alpha(1,1,\mathrm{GC})$. Therefore, the optimal GC does not depend on N in the neutral limit.

Single stability fitness function

To simplify the calculations and get more analytic insight into the model, we consider here a simplified fitness function that depends only on the smaller between the two stabilities, $\varphi = -\log(1+x^{-S})$ with $x = \min(x_\alpha, x_F)$. This fitness function is the neutral limit of Eq. (2) for $S \gg 1$. In fact, $\varphi = -\log\left[1+x^{-S}\left(1+(x_{\max}/x)^S\right)\right] \approx -\log(1+x^{-S})$. For this simplified model, the ML equation reads

$$\left[\frac{\partial\sigma}{\partial x}\right]_{x=\overline{x}} = -\frac{NS}{x(1+x^S)}\,, \tag{12}$$

and the Hessian becomes a scalar function $H = (\partial^2\sigma/\partial x^2) + N(\partial^2\varphi/\partial x^2) < 0$. We now compute how stability and fitness depend on S. For stability, we find

$$\frac{\partial\overline{x}}{\partial S} = -N\frac{1+\exp(-S\log(\overline{x})) - S\log(\overline{x})}{Hx\left(1+\overline{x}^S\right)^2}\,. \tag{13}$$

Since $H < 0$, the derivative is positive for $z < z^*$ and negative for $z > z^*$, where $z = S\log(\overline{x})$ and $z^* \approx 1.278$ is the non-trivial root of the equation $1 - z + \mathrm{e}^{-z} = 0$ at which stability reaches a maximum. Therefore, stability increases with the neutral exponent S for small S and it decreases for large S, consistent with the biological intuition. For fitness, we find

$$\frac{\mathrm{d}\varphi}{\mathrm{d}S} = \frac{\partial\varphi}{\partial S} + \frac{\partial\varphi}{\partial x}\frac{\partial\overline{x}}{\partial S} = \frac{S\log\overline{x} + \frac{S}{\overline{x}}\frac{\partial\overline{x}}{\partial S}}{\left(1+\overline{x}^S\right)}\,. \tag{14}$$

The denominator can be written as

$$\frac{\mathrm{d}\varphi}{\mathrm{d}S} \propto S\log\overline{x} - \frac{SN\left(1+\overline{x}^{-S} - S\log\overline{x}\right)}{H\overline{x}^2\left(1+\overline{x}^S\right)} = S\log\overline{x} + \frac{SN\left(1+\overline{x}^{-S} - S\log\overline{x}\right)\left(1+\overline{x}^S\right)}{NS\left(1+\overline{x}^S + S\overline{x}^S\right) + \left|\frac{\partial^2\sigma}{\partial x^2}\right|\left(1+\overline{x}^S\right)^2}\,. \tag{15}$$

For small S, it holds $\overline{x} \approx 0$ so that $S\log\overline{x} < 0$. The second term in the above equation is positive, but it is smaller than the first term since it is multiplied times S, hence the fitness decreases with S at very small S. At large S, $\log\overline{x}$ is positive and the fitness increases with S.

The previous analysis shows that, for fixed N and GC usage, there are two values of S at which two "evolutionary potentials" are extremal: The value of $S = S_{\mathrm{mut}}(N)$ at which the fitness is minimal, and the larger value $S = S_{\mathrm{opt}}(N)$ at which stability is maximal. This analysis defines in a natural way three regimes for the model, which are graphically represented in Fig. 1.

1. At small S, stability is close to the lethal threshold $\bar{x}=0$ where fitness drops to zero, purifying selection is weak and mutation entropy is large. We call this the mutation regime, and we define the mutation cross-over as the point where stability reaches the neutral threshold $\bar{x}=1$ at which the mean fitness takes the value $f=1/2$ that it has for $S=0$. We see from Eq. (12) that the boundary of the mutation regime is defined by the inequality

$$SN \leq -2\left[\frac{\partial\sigma}{\partial x}\right]_{x=1}. \tag{16}$$

Thus the larger is S, the smaller the populations size required to leave the mutation regime.

2. In the large S limit, the fitness becomes a binary variable with $f\approx 1$ for $x>1$ and $f\approx 0$ for $x<0$. In the neutral regime finite differences in stability imply very little differences in fitness and they cannot be fixed by natural selection, so that stability decreases with S towards the neutral threshold $\bar{x}=1$ for which mutation entropy is largest. We write the ML equation Eq. (12) in the form

$$1+\bar{x}^S = \frac{NS}{x\partial\sigma/\partial x} \approx \frac{NS}{|\partial\sigma/\partial x|_{x=1}}, \tag{17}$$

where we approximate $\bar{x}\approx 1$ in the r.h.s. At dominant order in S we obtain

$$\bar{x} \approx 1+\frac{1}{S}\log\left(\frac{NS}{|\partial\sigma/\partial x|_{x=1}}-1\right). \tag{18}$$

We now define the neutral cross-over as the point where $\bar{x}\leq 1+\varepsilon$, with small ε. From the above equations it follows that the neutral regime is defined through the inequality

$$N \leq \frac{1}{S}\left(e^{S\varepsilon}+1\right)\left|\frac{\partial\sigma}{\partial x}\right|_{x=1}. \tag{19}$$

Thus, the larger is S, the larger the population sizes N required to leave the neutral regime.

3. For N larger than the mutation cross-over (small S) or N larger than the neutral cross-over (large S) the population enters the non-neutral regime where stability is above the neutral threshold. For fixed N, stability has a maximum $x^*(N)$ at $S=S_{\mathrm{opt}}(N)$ given by

$$S_{\mathrm{opt}}(N) \approx \frac{1.278}{\log(x^*(N))}. \tag{20}$$

The above formulas suggest that the typical scale of population size for entering the non-neutral regime from the mutation or the neutral regime is proportional to $2\,|\partial\sigma/\partial x|_{x=1}$. We estimated this quantity from our simulations using the approximate relationship Eq. (18) to yield

$$-\left.\frac{\partial\sigma}{\partial x}\right|_{x=1} \approx \frac{NS}{1+\bar{x}^S}. \tag{21}$$

This estimate is predicted to be independent of N for very large S. We used data for $S=20$, finding that there is still some weak dependence on N that can be attributed to corrections to the above approximations. Averaging over N between 10 and 4000, we found that $2\,|\partial\sigma/\partial x|_{x=1}$ varies from a minimum of 28 at GC ≈ 0.5 to a maximum of 100 at GC ≈ 0, see Fig. 6. The minimum of $|\partial\sigma/\partial x|_{x=1}$ at GC ≈ 0.5 is another way to see that this mutation bias is optimal for neutral evolution.

Notice that there is a critical value of S that separates the mutation regime (small S) from the neutral regime (large S). This can be obtained by equating the boundaries of the two regimes, Eq. (16) and (19), which yields

$$2S^*+1 = e^{S^*\varepsilon}. \tag{22}$$

For $\varepsilon=0.5$ we find $S^*=1.63$. Similarly, there is a value of N below which the system passes directly from the mutation regime to the neutral regime without entering non-neutrality. This is given by $N=2/S^*\,|\partial\sigma/\partial x|_{x=0}$.

---

[1] Sella, G., Hirsh, A.E. (2005) The application of statistical physics to evolutionary biology. *Proc. Natl. Acad. Sci. USA* **102**:9541-9546.

[2] dos Reis, M., Savva R., Wernisch L. (2004). Solving the riddle of codon usage preferences: A test for translational selection, *Nucl. Ac. Res.* **32**:5036-5044.

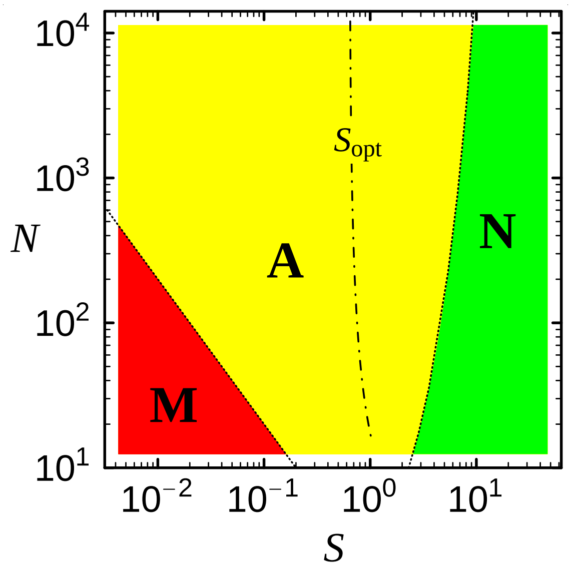

FIG. 1: Phase diagram showing the different regimes in the (N,S)-plane, with the border $S_{\rm mut} = 2/N$ between mutational and adaptive regime and with the border $S_{\rm neut} = \log(N)$ between the adaptive and neutral regime. The line $S_{\rm opt} = s^*/(2-3/\sqrt{N})$ with $s^* \approx 1.278$ being the non-trivial root of the equation $1 - s + e^{-s} = 0$ inside the adaptive regime displays the value of S for which for given N the fitness is maximum.

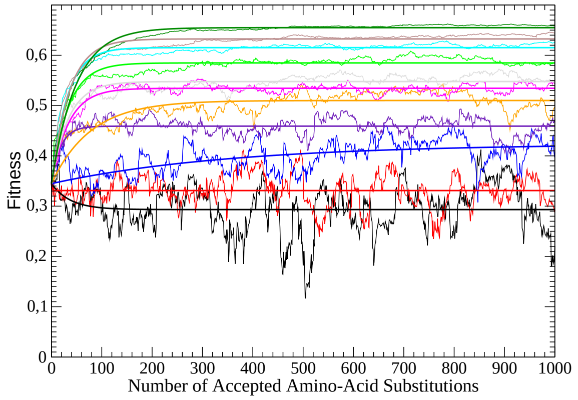

FIG. 2: Fitness versus number of accepted amino acid substitutions for different population sizes from 10 to 2000 and GC=0.5. One can see that the stationary distribution of fitness is reached in all cases after a number of substitutions of order of few times 100 for the protein lysozyme having 129 amino acids.

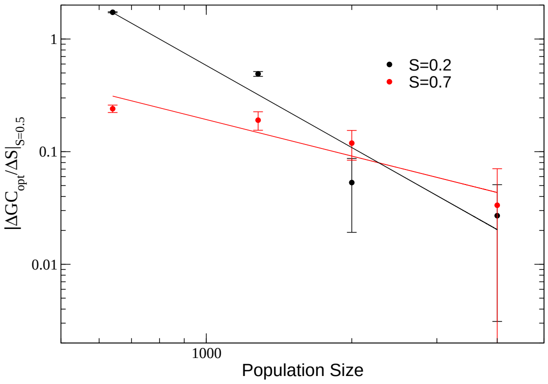

FIG. 3: Absolute value of the incremental ratio of the optimal GC bias as a function of S, $\left|\left(\mathrm{GC}_{\rm opt}(S) - \mathrm{GC}_{\rm opt}(S=0.5)\right)/(S-0.5)\right|$ versus population size N. The result is consistent with the expectation that $\mathrm{GC}_{\rm opt}$ becomes independent of S in the infinite population limit.

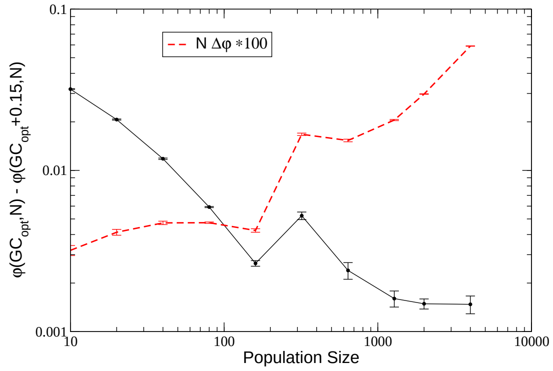

FIG. 4: Difference of selective coefficients $\Delta\varphi$ comparing the optimal GC usage and $\mathrm{GC_{opt}} - 0.15$. One can see that $\Delta\varphi$ decreases with population size N, however $N\Delta\varphi$ increases with N so that the optimal GC usage would be eventually selected even for large populations.

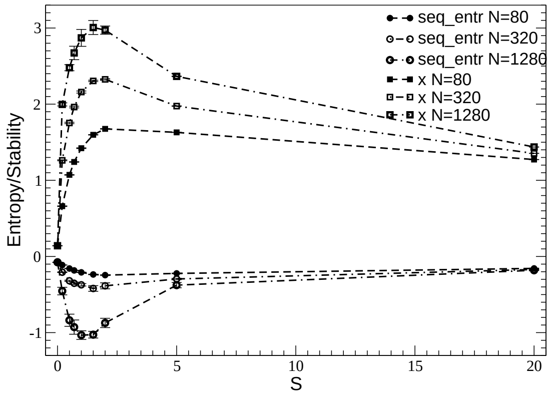

FIG. 5: Minimal stability $x = \min\{\alpha/\alpha_{\mathrm{thr}}, F/F_{\mathrm{thr}}\}$ and difference between actual sequence entropy and entropy expected under mutation alone, versus neutrality S for various population sizes and $\mathrm{GC} = 0.5$. Entropy was computed through the independent sites approximation as $\mathrm{Entropy} = -\sum_i \sum_a \mathrm{P}_i(a) \log(\mathrm{P}_i(a))$, where $\mathrm{P}_i(a)$ is the asymptotic distribution of amino acid a at site i. Entropy under mutation alone is computed using the distribution $\mathrm{P_{mut}}(a)$ in which each of the codons codifying the amino acid a have the frequency expected under the mutation model (with zero frequency for stop codons). The difference between the two entropies estimates the reduction in entropy due to negative selection for conserving protein stability. Notice that the maximum of stability corresponds to maximum reduction of sequence entropy.

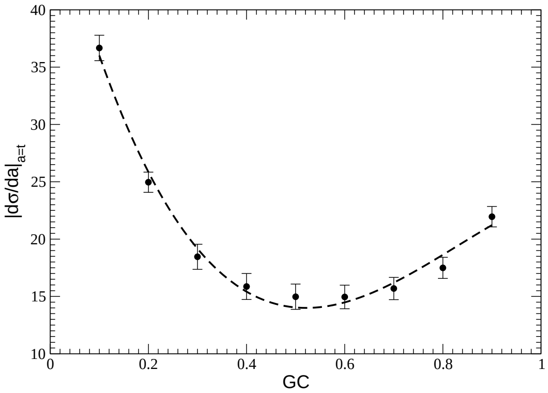

FIG. 6: Mutation bias dependence of $|\partial\sigma/\partial a|_{x=1}$ at the neutral threshold, obtained through Eq. (21) from simulations with $S = 20$, averaging over $N \in [10, 4000]$. The dashed line is a cubic interpolation that evidentiates the minimum at $\mathrm{GC} \approx 0.5$. This quantity sets the population size scale for passing to the non-neutral regime from the mutation regime or the neutral regime at $S \approx 1$.

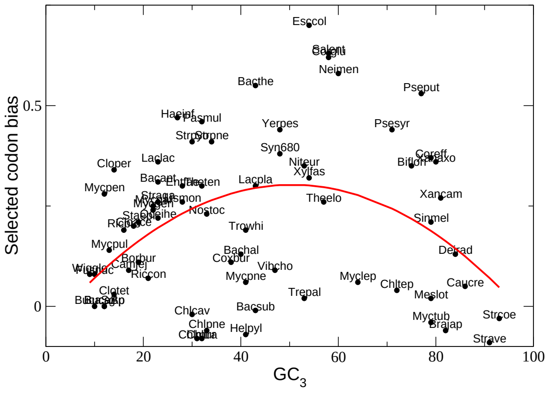

FIG. 7: Scatter plot of the selected optimal codon usage parameter computed by dos Reis *et al.* [2], which we use to estimate the effective population size, versus the GC content at third codon position. The line is a parabolic fit to the data.

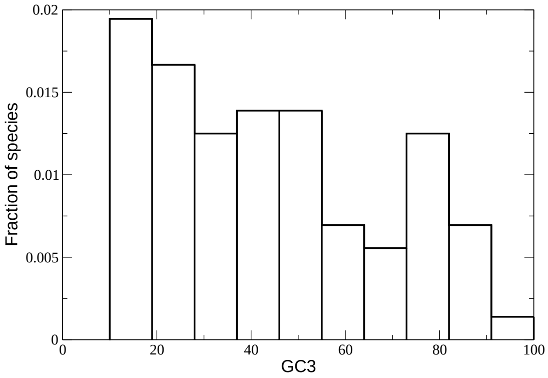

FIG. 8: Histogram of the GC content from the previous plot.
